# Cytogenetics of the true bug infraorder Cimicomorpha (Hemiptera, Heteroptera): a review

**DOI:** 10.3897/zookeys.154.1953

**Published:** 2011-12-12

**Authors:** Valentina G. Kuznetsova, Snejana M. Grozeva, Seppo Nokkala, Christina Nokkala

**Affiliations:** 1 Zoological Institute RAS, Universitetskaya emb. 1, St Petersburg 199034, Russia; 2 Institute of Biodiversity and Ecosystem research, BAS, Tsar Osvoboditel blvd, 1, Sofia 1000, Bulgaria; 3 Laboratory of Genetics, Department of Biology, University of Turku, 20500 Turku, Finland

**Keywords:** Hemiptera, Heteroptera, Cimicomorpha, holokinetic chromosomes, telomeres, NOR, chromosome number, m-chromosomes, sex chromosomes, B-chromosomes, meiosis

## Abstract

The Cimicomorpha is one of the largest and highly diversified infraorders of the Heteroptera. This group is also highly diversified cytogenetically and demonstrates a number of unusual cytogenetic characters such as holokinetic chromosomes; m-chromosomes; multiple sex chromosome systems; post-reduction of sex chromosomes in meiosis; variation in the presence/absence of chiasmata in spermatogenesis; different types of achiasmate meiosis. We present here a review of essential cytogenetic characters of the Cimicomorpha and outline the chief objectives and goals of future investigations in the field.

## Introduction

The Heteroptera, or true bugs, are a diversified group of insects displaying a number of unusual and sometimes unique cytogenetic characters such as holokinetic chromosomes, m-chromosomes, multiple sex chromosome systems, sex chromosome post-reduction and occasionally pre-reduction in male meiosis, variation in the presence/absence of chiasmata in spermatogenesis, different types of achiasmate meiosis and others. The pioneer investigators of true bug cytogenetics were Henking (1891), McClung (1902) and (Wilson (1905, 1909). It should be noticed that Hermann Henking (1891) and his object, the firebug *Pyrrhocoris apterus* Linnaeus, 1758 (Pentatomomorpha: Pyrrhocoridae), deserve the credit for the discovery of a relation between chromosomes and sex determination in animals. Since that time chromosomal sex determination has become more and more widely accepted among biologists.

The cytogenetics of the Heteroptera has been firstly comprehensively reviewed by Ueshima (1979) and shortly afterwards by Manna (1984). Ueshima’s (1979) superior monograph covers characteristics of all but one (Enicocephalomorpha, for which information is lacking to this day) heteropteran infraorders. However, the infraorders are cytogenetically unequally explored.

Since Ueshima’s publication a large body of new cytogenetic data on the Heteroptera has been obtained, including those on the cimicomorphan families Tingidae (Nokkala and Nokkala 1984a, Grozeva and Nokkala 2001), Anthocoridae s.str. (Nokkala and Nokkala 1986a, Wang et al. 2003), Microphysidae (Nokkala and Grozeva 2000), Cimicidae (Grozeva and Nokkala 2002, Poggio et al. 2009, Grozeva et al. 2010, 2011), Reduviidae (Pérez et al. 2004, Severi-Aguiar et al. 2006, Poggio et al. 2007, 2011, Panzera et al. 2010, Bardella et al. 2010 ), Nabidae s.str. (Nokkala and Nokkala 1984b, Kuznetsova and Maryańska-Nadahowska 2000, Kuznetsova et al. 2004, 2007, Kuznetsova and Grozeva 2008, Angus et al. 2008), and Miridae (Nokkala 1986a, Nokkala and Nokkala 1986b, Grozeva et al. 2006, 2007, 2011, Grozeva and Simov 2008a, b, Grozeva and Simov 2009). At present, the families Miridae and Reduviidae are the most extensively studied (data are available for 196 species in 83 genera and for 148 species in 45 genera, respectively), whereas the families Anthocoridae s.str. (5 species, 3 genera), Polyctenidae (3 species, 2 genera), Microphysidae (2 species, 2 genera), and the monospecific family Joppeicidae, are the least studied. In the three remaining families, data are available for 53 species (20 genera) in Cimicidae; 29 species (7 genera) in Nabidae s.str.; and 28 species (17 genera) in Tingidae (Table 1). At present, no cytogenetic data are available for the families Pachynomidae, Vianaididae (often included in the Tingidae), Velocipedidae and Medocostidae (both sometimes included in the Nabidae s.l.), Thaumastocoridae (possibly partly belonging to the Pentatomomorpha), Plokiophilidae, and Lasiochilidae and Lyctocoridae (prior to Schuh and Štys (1991), classified within Anthocoridae s.l.).

**Figure 1.**
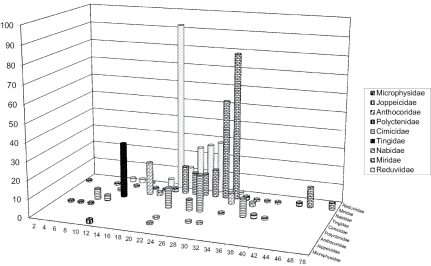
Autosome numbers’ range in Cimicomorpha. X-axis denotes the diploid number of autosomes, Y-axis shows the number of species

**Figure 2.**
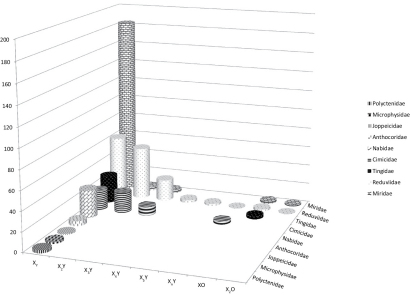
Distribution of sex chromosome systems in Cimicomorpha. Different sex chromosome systems are plotted on the X-axis. Y-axis shows the number of species. X_n_ - the number of X-chromosomes exceeds 5.

The Cimicomorpha is one of the largest and highly diversified heteropteran infraorders. Although this group has attracted considerable interest for several reasons (disease transmission in the Triatominae, evolution of host-plant relationships in the Miridae, maternal care in the Tingidae and so on; Schuh et al. 2009), cimicomorphan higher-level relationships are complex both at the family and tribal levels and subjected to several recent analyses (Schuh and Štys 1991, Schuh 1995, Schuh et al. 2009). Cytogenetically considered, Cimicomorpha appear likewise sufficiently heterogeneous. The aim of the present paper is to synthesize main data available concerning cytogenetic characteristics of cimicomorphan true bugs and to gain a better insight into the cytogenetic evolution within different families and the Cimicomorpha as a whole. A further aim is to outline the chief objectives and goals of future investigations in the field. The principle cytogenetic features of Cimicomorpha are summarized in Table 1 and in Figures 1 and 2.

## Holokinetic chromosomes and mechanisms of their evolution

Holokinetic chromosomes (sometimes designated as holocentric) occur in certain scattered groups of plants and animals, being particularly widespread in insects, including the Heteroptera (Kuznetsova et al. 2002, Lukhtanov and Kuznetsova 2009). These chromosomes have no primary constriction, the centromere, which is considered non-localized, or diffuse, formed by a large kinetochore plate extending all or most of the length of a chromosome (Schrader 1947, Wolf 1996).

Despite an important role of chromosomal change in the evolution and diversification of many groups of organisms (White 1978, King 1993, Coyne and Orr 2004, Ayala and Coluzzi 2005), the mechanisms behind this process are still little known, and this is especially true for groups with holokinetic chromosomes. Theoretically, the large kinetochore plate facilities karyotype evolution via occasional fusion/fission events. First, fused holokinetic chromosomes can not give rise to dicentric chromosomes. Second, any chromosome fragment exhibits a part of the kinetochore plate and can attach to spindle fibers at cell divisions. As a result, chromosome fragments that would be acentric (lacking a centromere) and hence lost in organisms with monocentric chromosomes (with localized centromeres) may be inherited in Mendelian fashion in holokinetic organisms, and gametes harbouring chromosome fragments are consequently expected to be viable (Hipp et al. 2010). Fusion/fission rearrangements are therefore conventionally accepted as the commonest mechanisms of chromosomal evolution in holokinetic groups. This assumption seems to receive support from the fact that the greatest range of within-genus karyotype variation related to the fusion/fission rearrangements is just described in organisms with holokinetic chromosomes. In metazoan animals, these are the blue butterfly genus *Agrodiaetus* Übner, 1822 and the gall inducing coccomorphan genus *Apiomorpha* Rübsaamen, 1894 in which diploid chromosome number ranges from 20 to 268 (Lukhtanov et al. 2005) and from 4 to ca. 192 (Cook 2000), respectively, whereas in plants – the angiosperm genus *Carex* Linnaeus, 1753 and the grass genus *Bromus* Linnaeus, 1753in which it varies from 12 to 132 (Hipp 2007) and from 14 to 105 (Joachimiak et al. 2001), respectively. Although variations in chromosome number of related species are probably due to both fissions and fusions of holokinetic chromosomes, fusions are suggested to be more common. The point is that a chromosome, be it holokinetic or monocentric, has to display two functional telomeres in order to survive a mitotic cycle. The fusion chromosome always displays functional telomeres originated from the ancestral chromosomes, whereas a fission chromosome has to be able to develop a functional telomere *de novo* (Nokkala et al. 2007).

## Chromosome numbers and modes of their transformation in Cimicomorpha

Chromosome numbers have been published for approximately 465 species (180 genera) of cimicomorphan true bugs, including many of the higher taxonomic categories within the infraorder (Table 1). In these species chromosome numbers range from 2n=6 in *Hesperoctenes fumarius* (Westwood, 1874) from thefamily Polyctenidae (Ueshima 1979) to 2n=80 in four species of the genus *Lopidea* Uhler, 1872 from the family Miridae (Akingbohungbe 1974). Cobben (1986) claimed to have found 2n=4 in *Hallodapus albofasciatus* (Motschulsky, 1863), Miridae, but this interpretation is not necessarily correct (this refers equally *Systellonotus alpinus* Frey-Gessner, 1871, 2n=8, in the same paper), since bivalents in the figures provided are clearly organized in a chain giving the false impression that the number of chromosomes is less than it is in reality (similarly as observed in *Piesma kochiae* (Becker, 1867); Grozeva 1991). It is worth noting that this range in chromosome number is larger than that reported for any other true bug infraorder, the number of 80 representing the highest one currently known in the Heteroptera as a whole. A number of cimicomorphan families demonstrate a considerably wide range of diploid chromosome numbers, the widest being in the families Miridae (from 14 to 80) and Cimicidae (from 10 to 50) (Table 1). These facts seemingly reinforce the fusion/fission hypothesis. However quite many cimicomorphan taxa show apparent karyotype conservation, with all or almost all species sharing the same chromosome number. This suggests that chromosomal fusions/fissions have played a minor role in the karyotype evolution and species diversification within these groups. By far the best example is the lace bug family Tingidae where all of 28 species studied (from 17 genera) have 12 autosomes in diploid complements differing only in sex chromosome system, which is XY or occasionally X0 in males (Nokkala and Nokkala 1984a, Grozeva and Nokkala 2001, Table 1). In other families chromosome number is more variable (Table 1, Fig. 1), and there is currently no obvious explanation of why karyotypes are less variable in the Tingidae than in the other cimicomorphan families. However in some within-family groups, for which a considerable body of information is amassed, chromosome number likewise appears remarkably stable, and modal (the commonest) chromosome numbers (at least autosome numbers) become obvious. The subfamilies Mirinae (Miridae) and Triatominae (Reduviidae) are a good case in point. In the Mirinae, the great majority of species have 2n=32+XY. In the Triatominae, which includes over 140 recognized species (in 15–19 genera), karyotypes are currently known for 84 (in 8 genera), and 80 of these species have 20 autosomes (Panzera et al. 2010, Table 1). Ueshima (1979) has suggested that this autosome number is plesiomorphic in the Triatominae and that fission and fusion rearrangements have resulted in the complements with 22 autosomes, as in *Triatoma rubrofasciata* (De Geer, 1773), and with 18 autosomes, as in *Triatoma nitida* Usinger, 1939 and *Panstrongylus megistus* (Burmeister, 1835).

However, the commonest chromosome number needs not to be plesiomorphic in a taxon. A good example comes from the family Nabidae s.str. In this relatively small family (20 genera and approximately 400 species), the number of autosomes reported for 29 species in 7 genera varies between 10 and 38 (Table 1). In addition to these values, there are also species with 16, 26, 30, and 32 autosomes. The predomination of the karyotype 2n=18(16+XY) discovered in 11 species and 4 genera has led to the hypothesis that it is the plesiomorphic condition in the family, and other chromosome numbers represent apomorphic characters (Leston 1957; Ueshima 1979, Thomas 1996, Kuznetsova and Maryańska-Nadachowska 2000). However, combining cytogenetic and karyosystematic knowledge (Kuznetsova et al. 2004, 2007) with a molecular phylogeny of the family based on 18S rDNA (Nokkala et al. 2007) provided conclusive evidence that the karyotype 2n=32+XY is plesiomorphic and the karyotype 2n=16+XY is apomorphic in the Nabidae s.str., and hence the evolution of karyotypes has been accompanied mainly by fusions of autosomes (Kuznetsova et al. 2004, 2007, Nokkala et al. 2007). In support of this conjecture one can argue that the high-number karyotypes, 2n=32+XY or close to it, appear also characteristic of the closely related families Miridae, Anthocoridae s.str., and Cimicidae (Table 1).

Considering the lack of a centromere, holokinetic chromosomes exhibit a very limited number of characters that can be used as markers. That is why, in spite of recent progress in developing of different staining techniques, chromosomal rearrangements not changing the number of chromosomes, such as inversions and reciprocal translocations, have been very rarely reported in the Heteroptera (Papeschi and Mola 1990, Bressa et al. 1998). Amongst Cimicomorpha, the triatomine species *Mepraia gajardoi* Frias, Henry and Gonzalez, 1998 provides an occasional example of a spontaneous translocation (Pérez et al. 2004). In a natural population of *Mepraia gajardoi*, a fusion between two non-homologous chromosomes was found in one of the eleven studied individuals. This autosomal translocation resulted in chromosomal irregularities such as an autosomal trivalent, autosomal univalents and chromosomal fragments, which altered the normal segregation of both autosomes and sex chromosomes. The extremely rare occurrence of translocations in the Triatominae led Pérez et al. (2004) to suggest that these structural rearrangements are strongly negatively selected, at least in this group.

## The m-chromosomes

The term “m-chromosomes” has been introduced by Wilson (1905) for a pair of very minute autosomes, which were first discovered in the coreid species *Anasa tristis* De Geer, 1773, in which these peculiar chromosomes behaved differently from both autosomes and sex chromosomes during male meiosis (Paulmier 1899). Thereafter m-chromosomes have been described in the karyotypes of many bug species (Ueshima 1979); however their origin and significance in genomes remain still obscure. As a rule, m-chromosomes are extremely small while in some species they might be of approximately the same size as the autosomes (Grozeva et al. 2009). Typically, m-chromosomes show negative heteropycnosis during meiotic divisions in males; they are unpaired during early meiotic prophase and hence form no chiasmata; they associate in a co-orientating pseudo-bivalent (the so-called a “touch-and-go” pairing) at metaphase I and segregate pre-reductionally at anaphase I (Ueshima 1979). However there are several observations suggesting that meiotic behavior of m-chromosomes is more complicated than has been understood earlier (cf. White 1973, Ueshima 1979). In *Coreus marginatus* Linnaeus, 1758 (Pentatomomorpha: Coreidae) the synapsis of m-chromosomes is shown to be quite normal at pachytene, suggesting that m-bivalents observed in a part of prophase cells are based on chiasma formation. Still m-chromosomes appear in a substantial part of prophase cells as univalents. In female meiosis, m-chromosomes form a chiasmate bivalent (Nokkala 1986b)

In Ueshima’s review (1979, p. 12) altogether 14 bug families are mentioned as having m-chromosomes, no cimicomorphan family being among them. Although in the recent reviews of Papeschi and Bressa (2006a, b) the Cimicomorpha is also referred as lacking m-chromosomes, they are however encountered sporadically among species in the families Miridae, namely, in *Adelphocoris lineolatus* (Goeze, 1778), *Dicyphus digitalidis* Josifov, 1958, *Deraeocoris rubber*Linnaeus, 1758, and *Deraeocoris rutilus* (Herrich-Schaeffer, 1838), *Capsus ater* (Linnaeus, 1758), *Dichrooscytus bureschi* Josifov 1969, *Lygus pratensis* (Linnaeus, 1758), *Notostira* *erratica* (Linnaeus, 1758) (Schachow 1932, Nokkala and Nokkala 1986b, Grozeva 2003, Grozeva and Simov 2008b, Grozeva et al. 2011), and Reduviidae, namely, in *Microtomus conspicillaris* Drury, 1782 and *Microtomus lunifer* (Berg, 1900) (Piza 1957, Poggio et al. 2011). The identification of m-chromosomes in the families with achiasmate type of male meiosis (see below) may be difficult, because in those meioses m-chromosomes always appear as a bivalent and not as univalents during meiotic prophase. Consequently, identification is based on the tiny size of a bivalent and the negative heteropycnosis it shows (Nokkala and Nokkala 1986b).

The currently available data suggest that the presence or absence of m-chromosomes represents a quite stable character at higher taxonomic levels in the Heteroptera, but only a few instances of the presence/absence of m-chromosomes in closely related true bug species have been reported (Ueshima 1979). In the Cimicomorpha, such examples are two mirid species, *Dicyphus albonasutus* Wagner, 1951 and *Dicyphus digitalidis* Josifov, 1958, the former lacking and the latter possessing m-chromosomes (Grozeva and Simov 2008b). However, the possibility can not be ruled out that in some cases m-chromosomes were not revealed because of their too small size and negative heteropycnosis in meiosis. The discovery of m-chromosomes in the basal infraorder Dipsocoromorpha (in the families Dipsocoridae and Schizopteridae)allowed the suggestion that m-chromosomes were present in the plesiomorphic karyotype of the Heteroptera (Grozeva and Nokkala 1996).

## Sex chromosome systems

Genetic sex determination is predominant in insects and is often accompanied by the presence of a heteromorphic chromosome pair in one sex. The true bugs share male heterogamety with the great majority of other insects. Within the Heteroptera, the XX/XY sex determination is of commonest occurrence, although XX/X0 and multiple sex chromosome systems (X_n_0, X_n_Y, and XY_n_) as well as rare neo-XY systems do occur (Ueshima 1979, Fig. 2).

The question as to whether the common ancestor of all Heteroptera was X0 or XY is still open. Ueshima (1979) has proposed that the XY system, despite its widespread occurrence in this group, is derived from the plesiomorphic X0 condition. The fact that sex determination in non-heteropteran Hemiptera groups is predominantly X0, the system being also considered plesiomorphic in Insecta as a whole (Blackman, 1995), seems to support this hypothesis.

On the other hand, (Nokkala and Nokkala (1983, 1984a) formulated an alternative hypothesis assuming that the XY mechanism is plesiomorphic in the Heteroptera, and the existence of X0 species is due to repeated loss of the Y chromosome, i.e. the result of convergent evolution (homoplasy). Their arguments are based on the discovery of an XY species, *Saldula orthochila* (Fieber, 1859), among X0 species in the genus *Saldula* Van Duzee, 1914 (Leptopodomorpha, Saldidae) and the sporadic occurrence of similar intrageneric X0-XY variation within the infraorders Gerromorpha, Cimicomorpha and Pentatomomorpha, indicating that the Y-chromosome has a tendency to get lost during evolution.

The most basal heteropteran infraorders are considered to be Enicocephalomorpha and Dipsocoromorpha (Wheeler et al. 1993, Schuh et al. 2009, Cassis and Schuh 2010). Unfortunately, in Enicocephalomorpha chromosomal data are still absent. In Dipsocoromorpha, such data are available for 2 species of the family Schizopteridae and for 4 species of the family Dipsocoridae, these species showing different sex chromosome systems, X0, XY and XY_1_Y_2 _(Ueshima 1979, Grozeva and Nokkala 1996). Moreover, within the genus *Pachycoleus* Fieber, 1860 both X0 species (*Pachycoleus rufescens* Sahlberg, 1875; Ueshima, 1979) and XY_1_Y_2_ species (*Pachycoleus pusillimus* (J. Sahlberg, 1870); Grozeva and Nokkala 1996: as *Cryptostemma* Herrich-Schaeffer, 1835) occur.

The existence of Y-chromosome in the Dipsocoromorpha seems to support the view that XY system evolved early in the evolution of the Heteroptera. Since the overwhelming majority of the true bug species possess Y chromosomes, the question arises about the origin of Y-chromosome in the Heteroptera. There is a variety of ways in which a Y-chromosome can evolve from an autosome (White 1973, Blackman 1995). One of those is a fusion between the X chromosome and an autosome (in an initially X0 species) resulting in a neo-XY system. In a recently formed neo-XY system, autosomally derived Y chromosome (a neo-Y) is still homologous with the autosome part of the neo-X and therefore synapses with it in meiosis. However the X and Y chromosomes in Heteroptera generally show little or no evidence of homology expected of a neo-XY system (Blackman 1995). Recently, a mechanism is revealed by which a heteropteran-like achiasmate Y-chromosome can evolve from a B-chromosome (supernumerary, or accessory, or extra chromosome; see below) (Nokkala et al. 2003, Nokkala and Nokkala 2004, Carvalho et al. 2009).

In the Cimicomorpha, the whole range of sex chromosome systems occurs. Within this infraorder, different sex chromosomes have evolved among closely related species or even intraspecific populations (Ueshima 1979, Panzera et al. 2010, Grozeva et al. 2010, Table 1, Fig. 2). However, species with the XY system clearly predominate. Amongst those families, in which the information is available on many species, the family Nabidae s.str. is the single one being exclusively XX/XY (Nokkala and Nokkala 1984b, Kuznetsova and Maryańska-Nadahowska 2000, Kuznetsova et al. 2004, 2007, Angus et al. 2008). Of the three most extensively studied families, X0 species are limited in Reduviidae and Miridae, and have never been reported in Cimicidae (Fig. 2, Table 1).

Compared to other Heteroptera, the Cimicomorpha is unique in that the majority of species posses multiple X chromosomes. Also, in this group the greatest number of X chromosomes in a species – up to 5 in the Reduviidae and up to 15 in the Cimicidae (Ueshima 1966a, b, 1979, Poggio et al. 2007, Grozeva et al. 2010, Table 1) is found. Within the Reduviidae, multiple sex chromosome systems are the most frequent in the subfamilies Harpactorinae and Stenopodinae (Poggio et al. 2007, 2011). In the Cimicidae, they are quite frequent in the subfamilies Cimicinae and Haematosiphoninae (Ueshima 1979, Poggio et al. 2009). One of the most intriguing examples is the bed bug *Cimex lectularius* Linnaeus, 1758 (Cimicidae), in which X chromosomes vary in number from two (X_1_X_2_Y) to 15 (X_1_X_2_Y+13 extra Xs) in different populations while sometimes between males of a population and even between different cells of a male (Ueshima 1966a, b, for other references see Grozeva et al. 2010). The origin of multiple systems in the Heteroptera is usually ascribed to simple transverse fissions of an original X chromosome, the process which is suggested to be facilitated by the holokinetic nature of true bug chromosomes (Schrader 1947, Ueshima 1966a, b, 1979). It is worth noting however that the application of C-banding to study the chromosomes of several Triatominae (Reduviidae) species led Panzera et al. (2010) to the conclusion that chromosomal rearrangements other than fissions might have been involved in the formation of the multiple sex chromosome systems in Heteroptera. However this problem clearly calls for further investigation.

## B-chromosomes

B-chromosomes, also known as supernumerary, accessory, or extra chromosomes, are dispensable elements which do not recombine with other chromosomes (the A-chromosomes) of the standard complement and follow their own evolutionary pathway (Beukeboom 1994). B-chromosomes are present in a part of individuals from some populations of a species resulting in intraspecific variation in chromosome number. The evolutionary significance of B-chromosomes seems to be evidenced by their widespread occurrence in very many plant and animal groups; however the origin, structure and evolution of these enigmatic chromosomes are still the subject of much controversy (Jones and Rees 1982, Camacho et al. 2000, Jones and Houben 2003, Camacho 2004). Within Cimicomorpha, B-chromosomes were described in 12 species, namely, *Triatoma longipennis* Usinger, 1939, *Mepraia gajardoi* Frias, Henry and Gonzalez, 1998, and *Mepraia spinolai* Porter, 1934 from the familyReduviidae ( Pérez et al. 2004, Panzera et al. 2010); *Orthocephalus funestus* Jakovlev 1881 from the Miridae (Takenouchi and Muramoto 1972b), *Acalypta parvula* (Fallén, 1807) and *Stephanitis oberti* (Kolenati, 1857) from the Tingidae (Grozeva and Nokkala 2001); *Nabis rugosus* (Linnaeus, 1758), *Nabis brevis* Scholtz, 1847, *Nabis ericetorum* Scholtz, 1847, and *Nabis pseudoferus* Remane, 1949 from the Nabidae s.str. (Grozeva and Nokkala 2003); *Paracimex borneensis* Usinger, 1959 and *Paracimex capitatus* Usinger, 1966 from the Cimicidae (Ueshima 1966b). The data obtained point to a sufficient variability of these supernumeraries in terms of their size, C-heterochromatin amount and distribution, meiotic behavior and impact on segregation of A-chromosomes in the species. By this is meant that B chromosomes in Cimicomorpha are of polyphyletic origin that correlates well with the modern concept of polyphyletic origin of B-chromosomes in different groups of animals and plants.

## Male meiosis

It is common knowledge that in meiosis, chiasmata (the points of genetic crossing-over) are formed uniting homologous chromosomes together until their separation in the reductional division. However in some animal groups chiasma formation is replaced by other, achiasmate means. When meiosis is achiasmate, at early prophase I one can see the conventional sequence of leptotene, zygotene and pachytene stages. However, no chiasmata are formed and hence no diplotene or diakinesis stages can be recognized. Typically, achiasmate meiosis is restricted to the heterogametic sex of a species. In most heteropteran males, autosomal bivalents are chiasmate whereas sex chromosomes have no chiasmata, however in a number of families male meiosis is completely achiasmate (Kuznetsova and Grozeva 2010). The first paper to describe the achiasmate meiosis within the Heteroptera was that of Nokkala and Nokkala (1983) dealing with the family Saldidae (Leptopodomorpha). Since that time, this meiotic pattern has been documented in six further heteropteran families, such as Micronectidae from Nepomorpha (Ituarte and Papeschi 2004, Grozeva et al. 2008) as well as Microphysidae, Nabidae s.str., Anthocoridae s.str., Cimicidae, and Miridae from Cimicomorpha (Nokkala and Nokkala 1984b, 1986a, b, Kuznetsova and Maryańska-Nadahowska 2000, Nokkala and Grozeva 2000, Grozeva and Nokkala 2002, Kuznetsova et al. 2004, 2007, Poggio et al. 2009, Grozeva et al. 2010, 2011). In Tingidae and Reduviidae, the remaining cimicomorphan families for which such evidence is available, males show the orthodox chiasmate meiosis. Nokkala and Nokkala (1984b) argued for a monophyletic origin of achiasmate meiosis in the Heteroptera. However, when more observations of achiasmate meiosis in Cimicomorpha and Nepomorpha became available, the polyphyletic origin of this type of meiosis in Heteroptera was suggested (Ituarte and Papeschi 2004, Grozeva et al. 2008).

Multiple origins of achiasmate meiosis in Heteroptera is substantiated by the placement of families with achiasmate meiosis in the cladogram based on combined analysis of 16S, 18S, 28S and COI sequence data and 73 morphological characters by Schuh et al. (2009, fig. 10). The existence of achiasmate meiosis in one family (Micronectidae) within Nepomorpha, in one family (Saldidae) within Leptopodomorpha, and in several families within Cimicomorpha is undoubtedly the result of independent events. Within the Cimicomorpha, the change from chiasmate to achiasmate meiosis could trace back to the separation of the clades Cimiciformes and Miroidea from the rest of the Geocorisae (node 12 in Schuh et al. 2009). All the families cytologically studied in the clade Cimiciformes show achiasmate male meiosis (Microphysidae, Nabidae s.str., Anthocoridae s.str., Cimicidae). In the sister clade Miroidea, the family Miridae shows achiasmate male meiosis, but male meiosis in the family Tingidae is chiasmate. According to this interpretation, achiasmate male meiosis in the Cimicomorpha is of monophyletic origin, and chiasmate meiosis in Tingidae represents reversal from achiasmate to chiasmate meiosis. An alternative explanation is that achiasmate meiosis has appeared coupled with the emergence of Cimiciformes and independently when the family Miridae was separated from their common ancestor with the Tingidae. In this alternative, achiasmate meiosis in the Cimicomorpha is of multiple origins and chiasmate meiosis in Tingidae is not of reversal type. As the latter alternative includes no reversal from achiasmate to chiasmate meiosis it seems more probable.

The multiple origin of achiasmate meiosis is well in accordance with the observations on the divergence in its cytological properties. The most common type of achiasmate meiosis is the so-called *alignment* type. In this type of meiosis, homologous chromosomes in a bivalent are held together along all their length during whole prophase up to metaphase I (Nokkala and Nokkala 1983). Within Cimicomorpha, the *alignment* type of meiosis has been described in the families Nabidae s.str. (Nokkala and Nokkala 1984b, Kuznetsova and Maryańska-Nadachowska 2000), Anthocoridae s.str. (Nokkala and Nokkala 1986a), and Microphysidae (Nokkala and Grozeva 2000). Beyond Cimicomorpha, this meiotic pattern is observed in both Saldidae (Nokkala and Nokkala 1983) and Micronectidae (Ituarte and Papeschi 2004, Grozeva et al. 2008).

In the *collochore* type, as it is called, one or occasionally two tenacious threads, the collochores, are formed to hold homologous chromosomes together in the absence of chiasmata. This pattern was described in the families Miridae and Cimicidae, in all the species studied in this respect (Nokkala and Nokkala 1986b, Grozeva and Nokkala 2002, Poggio et al. 2009, Grozeva and Simov 2008a, b, Grozeva et al. 2010, 2011, Grozeva and Simov 2009). Within the Miridae, the collochore meiosis is inherent in the genera *Bryocoris* Fallén, 1829 (1 species studied), *Dicyphus* Fieber, 1858 (10), and *Campyloneura* Fieber, 1858 (1), all of the subfamily Bryocorinae Baerensprung, 1860; *Deraeocoris* Kirschbaum, 1856 (2) from Deraeocorinae Douglas and Scott, 1865; *Capsus* Fabricius, 1803 (1) and *Megaloceroea* Fieber, 1858 (1) from MirinaeHahn, 1833; *Driophylocoris* Reuter, 1875 (2) from Orthotylinae Van Duzee, 1916); *Rhabdomiris* Wagner, 1968(1), *Pilophorus* Hahn, 1826 (1), *Plagiognathus* Fieber, 1858 (1), and *Cremnocephalus* Fieber, 1860 (2) from Phylinae Douglas and Scott, 1865). Within the Cimicidae, this meiotic pattern in inherent in the genera *Cimex* Linnaeus, 1758(3 species studied) from the subfamily Cimicinae Latreille, 1802, and in *Acanthocrios* Del Ponte and Riesel, 1945 (1), *Ornithocoris* Pinto, 1927 (1), and *Psitticimex* Usinger, 1966 (1) from the subfamily Haematosiphoninae Jordan and Rothschild, 1912.

Additionally, in the Nabidae s.str., where the tribes Nabini (Nabinae) and Prostemmatini (Prostematinae) are characterized by meiosis of the *alignment* type, a pattern intermediate between *alignment* and *collochore* meioses, has been described in *Arachnocoris trinitatus* Bergroth, 1916, the only representative of the tribe Arachnocorini Reuter, 1890 studied so far (Kuznetsova et al. 2007, Kuznetsova and Grozeva 2008).

In general, during the first division of meiosis the chromosomes reduce in number (reductional division), whereas during the second division the chromatids separate (equational division), and this pattern is named “pre-reduction” (White 1973). One of the unique cytogenetic characters of the Heteroptera, also presented in most taxa of Cimicomorpha, is the sex chromosome “post-reduction”, with sex chromosomes undergoing equational separation during first division and reductional segregation during second division. Autosomes always show the orthodox sequence of meiotic divisions in male meiosis. On occasion, individual bug species demonstrate sex chromosome pre-reduction, the Tingidae being the only heteropteran family showing pre-reductional behavior of sex chromosomes in spermatogenesis of all the species studied (Ueshima 1979, Grozeva and Nokkala 2003). The Tingidae are thus unique in having, besides sex chromosome pre-reduction, also unusually stable karyotype and chiasmate meiosis in males. It is interesting that all of these characters distinguish Tingidae from Miridae, the families considered to form a monophyletic group within Cimicomorpha (Schuh et al. 2009). In this infraorder, sex chromosome pre-reduction occurs likewise in all the three studied species of the genus *Macrolophus* Fieber, 1858 from Miridae (Grozeva et al. 2006, 2007) as well as in both studied species of the genus *Ectrychotes* Burmeister, 1835 from Reduviidae (Ueshima 1979, Manna and Deb-Mallick 1981), all other species of these families sharing sex chromosome post-reduction.

In most Cimicomorpha, as common in Heteroptera, sex chromosomes demonstrate the “*touch-and-go*” pairing at metaphase II of male meiosis, i.e. they come together forming a characteristic co-orientating pseudo-pair in the spindle and segregate polewards at anaphase II. The mechanism involved in this “*touch-and-go*” process (the term has been introduced by Wilson in 1925 for m-chromosomes demonstrating a similar behavior at metaphase I of meiosis) is a very puzzling one (Schrader 1940, Nokkala 1986a). The only exception presently known in Heteroptera is the subfamily Nabinae (Nabidae s.str.) where a kind of “*distance pairing”* of sex chromosomes at metaphase II is observed (Nokkala and Nokkala 1984b, Kuznetsova and Maryańska-Nadachowska 2000, Kuznetsova et al. 2004). Typical of *distance pairing* is that the sex chromosomes do not associate at metaphase II; they orientate towards opposite poles forming a kind of “*distance pseudo-pair*” and then segregate. It should be recorded that the other nabid subfamily, Prostemmatinae, shows the orthodox “*touch-and-go*” process (Kuznetsova et al. 2007).

Another characteristic feature is the configuration of metaphase I and metaphase II plates, which pattern seems to show species-specific variation in the Heteroptera (see Ueshima 1979). Meiotic metaphase plates in males are very often organized in such a way that both autosomal bivalents at MI and autosomes at MII form a circle in the center of which univalent chromosomes (X and Y chromosomes, m-chromosomes, B chromosomes) are placed. This configuration of the metaphase plate is referred to as radial as opposed to the other configuration, a nonradial one, where univalent chromosomes and autosomal bivalents are randomly distributed within the metaphase plate. The formation of radial metaphase plate is based on the congressional movements of bivalents and univalents that occur exceptionally along the nuclear envelope towards spindle equator during prometaphase I, resulting in both bivalents and univalents lying in a single ring at late prometaphase. Congression is followed by stabilization phase during which m-chromosome or sex chromosome univalents move along the equator to the center of the plate and form a co-oriented pseudo-bivalent at metaphase I or a pseudo-pair at metaphase II (“*touch-and-go*”) (Nokkala 1986a). Metaphases I and II or occasionally only one of them may be radial, closely related species sometimes differing in this pattern. In the families Nabidae s.str., Miridae, Microphysidae, and Anthocoridae s.str. the first metaphase plate is shown to be nonradial and the second metaphase plate radial (Nokkala and Nokkala 1984b, 1986a, b, Nokkala 1986a, Nokkala and Grozeva 2000, Kuznetsova et al. 2004). In the Cimicidae, *Cimex lectularius* demonstrates the same pattern (Grozeva et al. 2010), whereas in *Psiticimex uritui* Lent and Abalos, 1946, both MI and MII plates seem to be radial (see Figs 2b, c in Poggio et al. 2009). Typically, the stage between two meiotic divisions, interkinesis, is absent in spermatogenesis in the Heteroptera, and the first anaphase spindle is transformed directly into the second division spindle (Ueshima 1979). However, interkinesis stage is present in those taxa, where, as in Nabidae s.str. and Miridae, the first metaphase is nonradial and the second metaphase is radial. This stage is necessary for the formation of a radial metaphase II after a nonradial metaphase I (Nokkala and Nokkala 1984b, Nokkala 1986b).

## Female meiosis

For technical reasons, most research on heteropteran chromosomes has used males and as a consequence, there is very little evidence on meiosis in females. Helenius (1952; see also Piza 1957) was first to point out different orientation of autosomal metaphase I bivalents in male and female meiosis of the lygaeid bugs (Pentatomomorpha, Lygaeidae s.l.): in males parallel and in females perpendicular to the spindle axis. Based on this he claimed that meiosis in females was of the inverted type or post-reductional. On similar basis post-reduction was also suggested by Nokkala (1986a) in female meiosis of *Coreus marginatus* Linnaeus, 1758 (Pentatomomorpha, Coreidae). Later, however, it has been established that chiasma terminalization is absent in holokinetic chromosomes as evidenced by observations in *Triatoma infestans* (Klug, 1834) (Cimicomorpha, Reduviidae) (Pérez et. al.1997) and *Myrmus miriformis* (Fallen, 1807) (Pentatomomorpha, Rhopalidae) (Nokkala and Nokkala 1997). Consequently, the part of a half-bivalent extending from the chiasma point to the kinetic end separates pre-reductionally. Hence**,** chiasmate bivalents, irrespective of their orientation at metaphase I, always undergo pre-reduction (Nokkala and Nokkala 1997, Pérez et al. 1997, Nokkala et al. 2006, Viera et al. 2009) both in males and females.

One of the mirid species, *Campyloneura virgula* (Herrich-Schaeffer, 1835), is known to be mainly parthenogenetic, and males are extremely rare over the species distribution range (Wheeler 2001). A cytogenetic study of a parthenogenetic population from Samothraki (Northern Greece) has shown females to be diploid with the karyotype most characteristic of the family Miridae, i.e. 2n=32+XX. In these females, normal meiosis is suggested to be substituted by a modified mitotic division, and the oogenesis is hence of the apomictic type (Grozeva and Simov 2008b).

## Challenges and perspectives

In general, cytogenetic studies of the Heteroptera use standard techniques providing evidence on chromosome number, sex chromosome mechanisms and, in outline, the behavior of chromosomes during meiosis. For an investigator of true bug cytogenetics the basic challenge is the identification of individual chromosomes and chromosomal regions in a karyotype. This is just a condition under which the evolutionary rearrangements, both interchromosomal and intrachromosomal, could be detected in holokinetic chromosomes, that would result in considerable progress in the field. With differential cytogenetic techniques, only C-banding and DNA specific fluorochrome staining to reveal C-heterochromatin amount, distribution and composition, and NOR-staining to detect the number and location of nucleolus organizer regions (NORs) have been generally applied in the Heteroptera. However these approaches made possible only a few markers to be revealed in karyotypes. Nevertheless, they made it clear that taxonomically closely related species, even though they have the same chromosome number, do not in fact display identical karyotypes due to accumulation of many rearrangements since divergence from the common ancestor (Grozeva and Nokkala 2001, Angus et al. 2004, Grozeva et al. 2004, Kuznetsova et al. 2007). For example, the tribes in the family Nabidae s.str. were shown to differ in the location of NORs which are situated on sex chromosomes in Nabini (Nabinae) and Prostemmatini (Prostemmatinae) (Grozeva et al. 2004) and on a pair of large autosomes in Arachnocorini (Nabinae) (Kuznetsova et al. 2007).

In the last few decades, the ability to identify chromosomes has been markedly improved by the development of molecular cytogenetic technologies such as fluorescence *in situ* hybridization (FISH) for the mapping of genes and sequences, comparative genomic hybridization (CGH) for comparative analyses of genome homology, and others. Unfortunately, these useful approaches are not yet developed in the Heteroptera, with the sole exception of FISH with ribosomal probes to determine where ribosomal genes (18S, 28S or 45S) are located on the chromosomes of a species (Severi-Aguiar et al. 2006, Papeschi and Bressa 2006b, Grozeva et al. 2010, 2011, Panzera et al. 2010, Bardella et al. 2010, Poggio et al. 2011). Based on the very first data obtained we safely assume that molecular cytogenetic techniques will be beneficial for revealing additional chromosome markers and providing useful insight into the understanding of genome constitution and the mechanisms of karyotype evolution in true bugs. For example, in the family Reduviidae, FISH experiments using a 45S rDNA probe revealed differences in the number and location of hybridization sites between triatomine species sharing the same chromosome number, 2n=20+XY. In *Triatoma brasiliensis* Neiva, 1911 and *Triatoma rubrovaria* Blanchard, 1834, a single 45S rDNA cluster was found on a pair of autosomes, whereas in *Triatoma infestans melanosoma* Lent, Jurberg, Galvão and Carcavallo, 1994 on the X chromosome, while in *Triatoma matogrossensis* hybridization signals were located on both X and Y chromosomes (Bardella et al. 2010).

A potential ﬁeld of interest concerns the molecular composition of telomeres, which is totally unknown in the true bugs. Telomeres are terminal regions of chromosomes that protect chromosomes from destruction and stabilize their structure (Zakian 1995). DNA of the telomeric regions consists of short nucleotide motifs repeated thousands and millions of times. Comparative analysis of these motifs in various groups of organisms showed that they were evolutionarily stable, and mark taxa and phylogenetic branches of higher ranks (Traut et al. 1999). A pentanucleotide repetitive sequence, (TTAGG)_n_, is the commonest and most likely an ancestral telomeric motif of Insecta that supports their origin from a common ancestor. Heteroptera belong to a very few higher taxa of Insecta in which (TTAGG)_n_ telomeric sequence is absent as evidenced by FISH and/or Southern and/or dot-blot hybridization with a TTAGG probe (Sahara et al. 1999, Frydrychová et al. 2004, Grozeva et al. 2011). It is worthy of note that non-heteropteran Hemiptera, the Auchenorrhyncha included, retain this telomeric sequence (Frydrychová et al. 2004), however at present, data are not available for Colleorrhyncha, or moss bugs, widely considered to be the sister-group to Heteroptera. The (TTAGG)_n_ motif was suggested to be lost in the early evolution of the true bugs being secondarily replaced by another motif or an alternative telomerase-independent mechanism of telomere maintenance (Frydrychová et al. 2004, Lukhtanov and Kuznetsova 2010). Importantly, dot-blot hybridization of the genomic DNA from the true bug species with telomeric probes of different groups of animals and plants, namely, ciliate (TTTTGGGG)_n_ and (TTGGGG)_n_, nematode (TTAGGC)_n_, shrimp (TAACC)_n_, vertebrate (TTAGGG)_n_, and plant (TTTAGGG)_n_, yielded likewise negative results (Grozeva et al. 2011). On the basis of present knowledge, it may be inferred that telomere elongation is telomerase-independent in true bugs.

**Table 1.** Chromosome numbers and sex chromosome systems in Cimicomorpha (The systematics at superfamily and family level is after Schuh and Štys (1991); the systematics at subfamily level generally follows Maldonado (1990), Putshkov and Putshkov (1986–1989) and Weirauch (2008) for the Reduviidae; Schuh (1995) and Schuh (2011) for the Miridae; Kerzhner (1996) for the Nabidae s.str.; Schuh and Štys (1991) and Schuh and Slater (1995) for the rest of families)

**Taxa**			**2n (number of species)**	**References**
**Family**	**Subfamily**	**Genus (number of species studied)**		
**Superfamily** **Reduvioidea**				
**Reduviidae Latreille, 1807** (45/148) (genera/species studied)	Bactrodinae Stål, 1866 (1/1)	*Bactrodes* Stål, 1860 (1)	24+XY	Ueshima 1979
	Ectrichodiinae Amyot and Serville, 1843 (1/2)	*Ectrychotes* Burmeister, 1835 (2)	28+X0	Manna 1951, Manna and Deb-Mallick 1981
	Emesinae Amyot and Serville, 1843 (3/3)	*Bagauda* Bergroth, 1903 (1)	32+XY	Ueshima 1979
		*Barce* Stål, 1866 (1)	18+XY	Ueshima 1963b
		*Empicoris* Wolf, 1881 (1)	14+XY	Ueshima 1963b
	Hammacerinae Stål, 1859 (1/2)	*Microtomus* Illiger, 1807 (2)	26+2m+XY	Piza 1957, Poggio et al. 2011
	Harpactorinae Amyot and Serville, 1843 (18/35)	*Acholla* Stål, 1862 (2)	20+X_1_X_2_X_3_X_4_X_5_Y (1)	1909, 1910, Troedsson 1944
			n=16 (1)	Payne 1909, Montgomery 1901a
		*Apiomeris* Hahn, 1831 (5)	22+XY	Payne 1912, Ueshima 1979, Poggio et al. 2007
		*Arilus* Hahn, 1831 (1)	22+X_1_X_2_X_3_Y	*Arilus cristatus* (Linnaeus, 1763) (Montgomery 1901a, Payne 1909, Troedsson 1944: as *Prionidus* Uhler, 1886)
		*Cosmoclopius* Stål, 1866 (2)	24+X_1_X_2_X_3_Y	Poggio et al. 2007
		*Cydnocoris* Stål, 1866 (1)	24+X_1_X_2_Y	Dey and Wangdi 1988
		*Coranaus* Curtis, 1833 (1)	24+X_1_X_2_Y	Jande 1959a
		*Fitchia* Stål, 1859 (1)	24+X_1_X_2_Y	Payne 1909
		*Harpactor* Laporte, 1833 (2)	24+X_1_X_2_X_3_Y	Manna 1951, Banerjee 1958, Jande 1959b
		*Heniartes* Spinola, 1840 (1)	22+XY	Ueshima 1979
		*Lophocephala* Laporte, 1833 (1)	24+X_1_X_2_Y	Satapathy and Patnaik 1989
		*Polididus* Stål, 1858 (2)	10+XY (1)	Manna and Deb-Mallick 1981
			10+XY	*Polididus armatissimus* Stål, 1859 (Toshioka 1936, Banerjee 1958)
			12+XY	*Polididus armatissimus* (Jande 1960)
		*Pselliopus* Bergroth, 1905 (1)	24+X_1_X_2_X_3_Y	Payne 1912, Goldsmith 1916
		*Rhynocoris* Hahn, 1834 (3)	24+X_1_X_2_X_3_Y	Manna 1951, Banerjee 1958, Jande 1959b, Dey and Wangdi 1988, Satapathy and Patnaik 1989
		*Rocconota* Stål, 1859 (1)	24+X_1_X_2_Y	Payne 1909
		*Sinea* Amyot and Serville, 1843 (6)	24+X_1_X_2_X_3_Y (5) 20+X_1_X_2_X_3_X_4_X_5_Y (1)	Montgomery 1901a*, Payne 1909, 1912, Troedsson 1944, Manna 1951
		*Sycanus* Amyot and Serville, 1843 (2)	24+X_1_X_2_X_3_Y	Manna 1951, Jande 1959a
		*Velinus* Stål, 1865 (1)	24+X_1_X_2_X_3_Y	Toshioka 1933, Yoshida 1947
		*Zelus* Fabricius, 1802 (2)	24+XY	*Zelus exsanguis* Stål, 1862 (Payne 1909: as *Diplacodus* Kirkaldy, 1900)
			24+XY	Poggio et al. 2007
	Peiratinae Stål, 1859 (4/6)	*Androclus* Stål, 1863 (1)	20+XY	Jande 1959b
		*Ectomocoris* Mayr, 1865 (3)	20+XY (1) 20+X_1_X_2_Y (2)	Jande 1959a
		*Rasahus* Amyot and Serville, 1843 (1)	20+X_1_X_2_Y	Ueshima 1979
		*Sirthenea* Spinola, 1837 (1)	26+XY	Jande 1959b
	Phymatinae Laporte, 1832 (2/2)	*Macrocephalus* Swederus, 1787 (1)	26+XY	Ueshima 1979
		*Phymata* Latreille, 1802 (1)	26+XY	Montgomery 1901a*
	Reduviinae (3/4)	*Pasiropsis* Reuter, 1881 (1)	24+X_1_X_2_X_3_X_4_Y	Jande 1959b
		*Reduvius* Fabricius, 1775 (2)	20+XY (1) 26+XY (1)	Payne 1912, Ueshima 1979 Ueshima 1979
		*Staliastes* Kirkaldi, 1900 (1)	24+XY	Jande 1959a
	Stenopodinae Amyot and Servielle, 1843 (4/11)	*Oncocephalus* Klug, 1830 (7)	20+X_1_X_2_Y (4) 20+X_1_X_2_X_3_Y (1) 22+X_1_X_2_X_3_Y (2)	Jande 1959a, Ueshima 1979, Manna and Deb-Mallick 1981, Satapathy and Patnaik 1989
		*Pnirontis* Stål, 1859 (1)	20+X_1_X_2_X_3_X_4_Y	Payne 1912
		*Pygolampis* Germar, 1817 (2)	22+XY (1) 22+X_1_X_2_Y (1)	Banerjee 1958 Jande 1959a
		*Stenopoda* Laporte, 1833 (1)	20+X_1_X_2_X_3_X_4_Y	Poggio et al. 2007
	Triatominae Jeannel, 1919 (8/84)	*Belminus* Stäl, 1859 (2)	20+X_1_X_2_Y	Panzera et al. 2010
		*Dipetalogaster* Usinger, 1939 (1)	20+XY	Ueshima 1966a
		*Eratyrus* Stäl, 1859 (2)	20+X_1_X_2_Y	Panzera et al. 2010
		*Mepraia* Mazza, Gajardo and Jörg, 1940 (3)	20+X_1_X_2_Y	Pérez et al. 2004, Frìas-Lasserre 2010
		*Panstrongylus* Berg, 1879 (9)	18+X_1_X_2_Y (1) 20+X_1_X_2_Y (7)	Ueshima 1966a, Schreiber et al. 1972, Panzera et al. 2010
			18+X_1_X_2_Y (1)	*Panstrongylus megistus* (Burmeister, 1835) (Schreiber and Pellegrino 1950, Barth 1956: as *Mestor* Kirkaldi, 1904)
		*Paratriatoma* Barber, 1938 (1)	20+XY (1)	Ueshima 1966a
		*Rhodnius* Stål, 1859 (15)	20+XY	*Rhodnius coreodes* (Bergroth 1911) (Schreiber and Pellegrino 1950: *as Psammolestes* Bergroth, 1911), Barth 1956, Ueshima 1966a, Petitpierre 1996, Panzera et al. 2010
		*Triatoma* Laporte, 1832 (51)	18+X_1_X_2_Y (1) 20+XY (25) 20+X_1_X_2_Y (21) 20+X_1_X_2_X_3_Y (2)	Schreiber and Pellegrino 1950, Barth 1956, Ueshima 1966a, Bargues et al. 2006; Panzera et al. 1995, 2004, 2006, 2010
			20+X_1_X_2_Y	*Triatoma sanguisuga* (LeConte, 1855)(Payne 1909, Panzera et al. 2010: as *Conorhinus* Laporte, 1832)
			22+X_1_X_2_Y	*Triatoma rubrofasciatus* (De Geer, 1773) (Manna 1950, 1951, Panzera et al. 2010: as *Conorhinus*)
**Superfamily Microphysoidea**				
**Microphysidae Dohrn, 1859** (2/2)		*Myrmedobia* Bärensprung, 1857 (1)	12+XY	Nokkala and Grozeva 2000
		*Loricula* Curtis, 1833 (1)	12+XY	*Loricula pselapfiformis* Curtis, 1833 (Grozeva, unpublished)
**Superfamily Joppeicoidea**				
**Joppeicidae Reuter, 1910** (1/1)		*Joppeicus* Putton, 1881 (1)	22+XY	Ueshima 1979
**Superfamily Miroidea**				
**Miridae Hahn, 1833** (83/196)	Bryocorinae Baerensprung, 1860 (9/27)	*Bryocoropsis* Schumacher, 1917 (1)	32+XY	Kumar 1971
		*Bryocoris* Fallén, 1829 (1)	32+XY	Grozeva and Simov 2008b
		*Campyloneura* Fieber, 1858 (1)	32+XX (♀♀)	Grozeva and Simov 2008b
		*Dicyphus* Fieber, 1858 (15)	36+XY (1) 40+XY (1) 44+XY(1) 44+X_1_X_2_Y (1) 46+XY (8) 46+2m+X_1_X_2_X_3_Y (1) 46+X_1_X_2_Y (1) 46+X_1_X_2_X_3_Y (1)	Slack 1938b*, Leston 1957, Grozeva 2003, Grozeva and Simov 2008b
		*Distantiella* China, 1944 (1)	26+XY	Kumar 1971
		*Helopeltis* Signoret, 1858 (2)	16+XY (1) 18+XY (1)	Kumar 1971
		*Macrolophus* Fieber, 1858 (3)	24+XY (1) 24+X_1_X_2_X_3_Y (1) 26+XY (1)	Grozeva et al. 2006, 2007
		*Molanocoris* Dahlbom, 1851(2)	32+XY	Slack 1938b, Leston 1957, Kumar 1971, Akingbohungbe 1974*, Grozeva and Simov 2008b
		*Sahlbergella* Haglund, 1895 (1)	26+XY	Kumar 1971
	Deraeocorinae Douglas and Scott, 1865 (2/17)	*Deraeocoris* Kirschbaum, 1856 (15)	32+XY (11) 34+XY (1) 30+2m+XY (2)	Southwood and Leston 1959*, Takenouchi and Muramoto 1968, Akingbohungbe 1974*, Grozeva et al. 2011
			32+XY	*Deraeocoris lutescens* Schilling, 1837 (Leston 1957: as *Camptobrochis* (Schilling, 1837))
		*Hyaliodes* Reuter, 1876 (2)	32+XY (1) 34+XY (1)	Akingbohungbe 1974*
	Mirinae Hahn, 1833 (34/73)	*Adelphocoris* Reuter, 1896 (7)	20+XY (1) 22+XY (2) 24+XY (1) 26+XY (1)	Takenouchi and Muramoto 1967, 1972a, b, Muramoto 1973a, Akingbohungbe 1974*
			12+2m+XO	*Adelphocoris lineolatus* (Goeze, 1778) (Schachow 1932*)
			30+XY	*Adelphocoris lineolatus* (Ekblom 1941: as /Calocoris chenopodii /Westhoff, 1881, Leston 1957, Akingbohungbe 1974*)
			2n=28	*Adelphocoris rapidus* (Say, 1832) (Akingbohungbe 1974*: as *Calocoris rapidus* Say, 1832)
			26+X_1_X_2_0	*Adelphocoris rapidus* (Mongomery 1901a, 1906: as *Calocoris* *rapidus*)
		*Apolygus* China, 1941 (3)	32+XY	Leston 1957, Muramoto 1973a
		*Camptozygum* Reuter, 1896 (1)	32+XY	Southwood and Leston 1959*
		*Capsus* Fabricius, 1803 (1)	32+XY	*Capsus ater* (Linnaeus, 1758)(Slack 1938b*, Akingbohungbe 1974*)
			30+2m+XY	*Capsus ater* (Nokkala and Nokkala 1986b)
		*Charagochilus* Fieber, 1858 (1)	32+XY	Muramoto 1973a
		*Closterotomus* Fieber, 1858 (2)	32+XY (1)	*Closterotomus fulvomaculatus* De Geer, 1773 (Slack 1938b*: as *Calocoris* Fieber, 1858)
			30+XY	*Closterotomus norvegicus* Gmelin, 1790 Slack 1938b*: as *Calocoris*)
			32+XY	*Closterotomus norvegicus* (Leston 1957: as *Calocoris*)
		*Collaria* Provancher, 1872 (1)	12+XY	Akingbohungbe 1974*
		*Creontiades* Distant, 1883 (1)	30+XY	Takenouchi and Muramoto 1970
		*Cyphodemidea* Reuter, 1903 (1)	32+XY	*Creontiades saundersi* Reuter, 1896 (Takenouchi and Muramoto 1967: as *Orthops*)
		*Dichrooscytus* Fieber, 1858 (2)	32+XY (1)	Akingbohungbe 1974*
			28+2m+XY (1)	Grozeva 2003
		*Eurystylus* Stål, 1871 (1)	30+XY	Takenouchi and Muramoto 1968
		*Garganus* Stål, 1862 (1)	32+XY	Akingbohungbe 1974*
		*Grypocoris* Douglas and Scott, 1868 (1)	30+XY	*Grypocoris sexguttatus* (Fabricius, 1777) (Slack 1938b*: as *Calocoris*)
		*Horcias* Distant, 1884 (1)	32+XY	Montgomery 1901a, 1906, Akingbohungbe 1974*
		*Leptopterna* Fieber, 1858 (1)	32+XY	Montgomery 1901a, Slack 1938b*, Leston 1957, Akingbohungbe 1974*
		*Liocoris* Fieber, 1858 (1)	32+XY	*Liocoris tripustulatus* (Fabricius, 1781) (Leston 1957: as *Lygus*)
		*Litomiris* Slater, 1956 (1)	32+XY	Akingbohungbe 1974*
		*Lygocoris* Reuter, 1875 (11)	32+XY (10)	Geitler 1939*, Leston 1957, Southwood and Leston 1959*, Muramoto 1973a, Akingbohungbe 1974*
			32+XY	*Liocoris rubripes* Jakovlev, 1876 (Takenouchi and Muramoto 1970: as *Adelphocoris*)
		*Lygus* Hahn, 1833 (5)	32+XY (4)	Leston 1957, Southwood and Leston 1959*, Akingbohungbe 1974*, Ueshima 1979
			n=19?	*Lygus pratensis* (Linnaeus, 1758) (Montgomery 1901a, 1906)
			30+2m+X0	*Lygus pratensis* (Schachow 1932*)
			32+XY	*Lygus pratensis* (Geitler 1939*)
		*Megacoelum* Fieber, 1858 (1)	30+XY	Leston 1957
		*Megaloceroea* Fieber, 1858 (1)	32+XY	*Megaloceroea recticornis* (Geoffroy, 1785) (Leston 1957)
			30+XY	*Megaloceroea recticornis* (Grozeva et al. 2011)
		*Neurocolpus* Reuter, 1876 (3)	32+XY	Akingbohungbe 1974*
		*Notostira* Fieber, 1858 (1)	12+2m+X0	Schachow 1932
		*Onomaus* Distant, 1904 (1)	30+XY	Takenouchi and Muramoto 1968
		*Orthops* Fieber, 1858 (1)	22+XY	*Orthops campestris* (Linnaeus, 1758) (Muramoto 1973a)
			32+XY	*Orthops campestris* (Akingbohungbe 1974*)
		*Phytocoris* Fallén, 1814 (9)	32+XY (8) 30+XY (1)	Slack 1938b, Leston 1957, Southwood and Leston 1959*, Akingbohungbe 1974*
		*Poecilocapsus* Reuter, 1876 (1)	32+XY	Montgomery 1901a, Akingbohungbe 1974*
		*Polymerus* Hahn, 1831 (3)	32+XY	Leston 1957, Akingbohungbe 1974*
		*Rhabdomiris* Wagner, 1968	30+XY	Nokkala and Nokkala 1986b: as *Calocoris quadripunctatus* (Villers, 1789)
		*Stenodema* Laporte, 1833 (2)	30+XY (1) 32+XY (1)	Leston 1957 Slack 1938b*, Leston 1957
		*Stenotus* Jakovlev, 1877 (2)	40+XY (1)	Takenouchi and Muramoto 1967
			30+XY	*Stenotus binotatus* (Fabricius 1794) (Slack 1938b*, Takenouchi and Muramoto 1967)
			32+XY	*Stenotus binotatus* (Leston 1957)
			2n=32-34	*Stenotus binotatus* (Akingbohungbe 1974*)
		*Taedia* Distant, 1883 (1)	32+XY	Akingbohungbe 1974*
		*Trigonotylus* Fieber, 1858 (2)	32+XY	Takenouchi and Muramoto 1967, Akingbohungbe 1974*
		*Tropidosteptes* Uhler, 1878 (1)	32+XY	Akingbohungbe 1974*
	Orthotylinae Van Duzee, 1916 (16/33)	*Brepharidopterus* Kolenati, 1845 (1)	22+XY	Leston 1957
		*Ceratocapsus* Reuter, 1876 (4)	18+XY (1) 22+XY (3)	Akingbohungbe 1974*
		*Cyllecoris* Hahn, 1834 (1)	21+ X_1_X_2_Y	Southwood and Leston 1959*
		*Dryophilocoris* Reuter, 1875 (2)	32+XY (1)	Grozeva and Simov 2011
			32+XY	*Dryophilocoris flavoquadrimaculatus* (De Geer, 1773) (Grozeva and Simov 2011)
			34+X_1_X_2_Y	*Dryophilocoris flavoquadrimaculatus* (Leston 1957)
		*Halticus* Hahn, 1832 (1)	28+XY	Akingbohungbe 1974*
		*Heterotoma* Lepeletier and Serville, 1825 (1)	32+XY	Southwood and Leston 1959*
		*Ilnacora* Reuter, 1876 (2)	24+XY	Akingbohungbe 1974*
		*Labops* Burmeister, 1835 (1)	38+XY	Akingbohungbe 1974*
		*Lopidea* Uhler, 1872 (4)	78+XY	Akingbohungbe 1974*
		*Malacocoris* Fieber, 1858 (1)	20+XY (1)	Leston 1957
		*Orthocephalus* Fieber, 1858 (2)	26+XY	Takenouchi and Muramoto 1967
			28+XY	Takenouchi and Muramoto 1972b, Muramoto 1973b, 1974
		*Orthotylus* Fieber, 1858 (7)	22+XY (2) 24+XY (1) 26+XY (2) 28+XY (1)	Slack 1938b*, Leston 1957, Southwood and Leston 1959*, Akingbohungbe 1974*
			24+XY	*Orthotylus flavosparsus* (C Sahlberg, 1841) (Leston 1957)
			26+XY	*Orthotylus flavosparsus* (Akingbohungbe 1974*)
		*Parthenicus* Reuter, 1876 (1)	22+XY	Akingbohungbe 1974*
		*Pseudoxenetus* Reuter, 1909 (1)	28+XY	Akingbohungbe 1974*
		*Reuteria* Puton, 1875 (1)	24+XY	Akingbohungbe 1974*
		*Slaterocoris* Wagner, 1956 (3)	24+XY	Akingbohungbe 1974*
	Phylinae Douglas and Scott, 1865 (22/46)	*Amblytylus* Fieber, 1858 (1)	30+XY	Southwood and Leston 1959*, Akingbohungbe 1974*
		*Atractotomus* Fieber, 1858 (1)	30+XY	Southwood and Leston 1959*
		*Campylomma* Reuter, 1878 (1)	30+XY	Akingbohungbe 1974*
		*Chlamydatus* Curtis, 1833 (4)	30+XY	Slack 1938b*, Leston 1957, Southwood and Leston 1959*, Akingbohungbe 1974*
		*Compsidolon* Reuter, 1899 (1)	30+XY	*Compsidolon beckeri* Reuter, 1904 (Akingbohungbe 1974*: as *Psallus*)
		*Conostethus* Fieber, 1858 (1)	30+XY	Slack 1938b*
		*Cremnocephalus* Fieber, 1860 (2)	26+XY (1) 28+XY (1)	Grozeva and Simov 2008a
		*Criocoris* Fieber, 1858 (1)	30+XY	Akingbohungbe 1974*
		*Europiella* Reuter, 1909 (1)	30+XY	*Europiella albipennis* (Fallén, 1829) (Leston 1957: as *Plagyognathus*)
		*Harpocera* Curtis, 1838 (1)	32+XY	Grozeva, 2003
		*Hallodapus* Fieber, 1858 (2)	24+XY (1) 2n=4 (1)**	Muramoto 1973a Cobben 1986
		*Lopus* Hahn, 1831(1)	30+XY	Slack 1938b*
		*Macrotylus* Fieber, 1858 (2)	32+XY	Geitler 1939*, Southwood and Leston 1959*
		*Megalocoleus* Reuter, 1890 (2)	30+XY	Leston 1957
		*Oncotylus* Fieber, 1858 (1)	30+XY	Leston 1957
		*Orectoderus* Uhler, 1890 (1)	32+XY	Akingbohungbe 1974*
		*Phoenicocoris* Reuter, 1875 (1)	30+XY	*Phoenicocoris rostratus* Knight, 1923 (Akingbohungbe 1974*: as *Lepidopsallus* Knight, 1923)
		*Phylus* Hahn, 1831(1)	30+X0	Leston 1957
		*Pilophorus* Hahn, 1826 (4)	26+XY (1) 28+XY (3)	Muramoto 1973a, Akingbohungbe 1974*
		*Plagiognathus* Fieber, 1858 (9)	30+X0 (1) 30+XY (4) 32+XY (1)	Slack 1938b*, Leston 1957, Takenouchi and Muramoto 1967, 1968
			32+XY (1)	*Phoenicocoris longirostris* (Knight, 1923) (Akingbohungbe 1974*:as *Microphylellus* Reuter, 1909)
			28+XY	*Phoenicocoris chrysanthemi* (Wolf, 1804) (Slack 1938b*, Akingbohungbe 1974*)
			30+XY	*Phoenicocoris chrysanthemi* (Leston, 1957)
			32+XY	*Phoenicocoris arbustorum* (Fabricius, 1794) (Slack 1938b*)
			30+XY	*Phoenicocoris arbustorum* (Leston 1957)
		*Psallus* Fieber, 1858 (7)	28+XY (3) 30+XY (4)	Slack 1938b*, Southwood and Leston 1959*, Takenouchi and Muramoto 1968, Muramoto 1973a
		*Systellonotus* Fieber, 1958 (1)	2n=8**	Cobben 1986
**Tingidae Laporte, 1832** (17/28)	Tinginae Laporte, 1832 (17/28)	*Acalypta* Westwood, 1840 (3)	12+X0 (2)	Grozeva and Nokkala 2001
			10+XY	*Acalypta parvula* (Fallén, 1807) (Southwood and Leston 1959*)
			12+X0	*Acalypta parvula* (Grozeva and Nokkala 2001)
		*Agramma* Stephens, 1829 (1)	12+XY	Muramoto 1973c
		*Cochlochila* Stål, 1873 (1)	12+XY	Takenouchi and Muramoto 1967
		*Copium* Thunberg, 1822 (1)	12+XY	Grozeva and Nokkala 2001
		*Corythucha* Stål, 1873 (1)	12+XY	Grozeva and Nokkala 2001
		*Cysteochila* Stål, 1873 (1)	12+XY	Jande 1960 ( as *Bredenbachius* Distant, 1903)
		*Dasytingis* Drake and Poor, 1936 (1)	12+XY	*Dasytingis bengalana* Drake, 1956 (Jande 1960: as *Tingis* Fabricius, 1803)
		*Dictyla* Stål, 1874 (2)	12+XY	*Dictyla humuli* (Fabricius, 1794) (Southwood and Leston 1959*: as *Monanthia* Lepeletier and Serville, 1828), Grozeva and Nokkala 2001
		*Dictyonota* Curtis, 1827 (1)	12+XY	Southwood and Leston 1959*
		*Elasmotropis* Stål, 1874 (1)	12+XY	Grozeva and Nokkala 2001*
		*Kalama* Puton, 1876 (1)	12+XY	Grozeva and Nokkala 2001
		*Lasiacantha* Stål, 1873 (1)	12+XY	Grozeva and Nokkala 2001
		*Leptobyrsa* Stål, 1873 (1)	12+XY	Harley and Kassulke 1971
		*Physatocheila* Fieber, 1844 (1)	12+XY	Grozeva and Nokkala 2001
		*Stephanitis* Stål, 1873 (3)	12+XY	*Stephanitis takeyai* Drake and Maa, 1955 (Toshioka 1934, Jande 1960: as *Monathia globulifera* Matsumura, 1905), Grozeva and Nokkala 2001
		*Teleonemia* Costa, 1864 (2)	12+XY	Harley and Kassulke 1971
		*Tingis* Fabricius, 1803 (6)	12+XY	Montgomery 1901a, 1906, Southwood and Leston 1959*, Muramoto 1973c*, Grozeva and Nokkala 2001
**Superfamily Naboidea**				
**Nabidae Costa A, 1853** (7/29)	Nabinae (5/27)	*Arachnocoris* Scott, 1881 (1)	10+XY	Kuznetsova et al. 2007, Kuznetsova and Grozeva 2008
		*Himacerus* s. str.Wolff, 1811 (1)	36+XY	*Himacerus apterus* (Fabricius, 1798) (Yoshida 1950, Kuznetsova et al. 2004, Angus et al. 2008)
			16+XY	*Himacerus apterus* (De Meijere 1930*, Leston 1957)
			38+XY	*Himacerus apterus* (Takenouchi and Muramoto 1968)
		*Himacerus* (*Aptus* Hahn, 1831) (2)	32-36+XY (1)	*Himacerus maracandicus* (Reuter, 1890) (Kuznetsova and Maryańska-Nadachowska 2000)
			32+XY (1)	*Himacerus mirmicoides* (O. Costa, 1834) (Leston 1957 Kuznetsova et al. 2004, Angus et al. 2008)
		*Himacerus* (*Stalia*) Reiter, 1872 (1)	30+XY	*Himacerus major* (A. Costa, 1841) (Angus et al. 2008: as *Stalia* Reuter, 1872)
		*Hoplistoscelis* Reuter, 1890 (1)	16+XY	Kuznetsova and Maryańska-Nadachowska 2000
		*Lasiomerus* Reuter, 1890 (1)	16+XY	Montgomery 1901b
		*Nabis* s.l. Latreille, 1802 (20)		
		*Nabis* s.str. (8)	16+XY (6)	Schachow 1932, Mikolajski 1964, 1965, 1967, Takenouchi and Muramoto 1967, 1968, 1969, Muramoto 1978, 1979, Nokkala and Nokkala 1984b, Kuznetsova and Maryańska-Nadachowska 2000, Grozeva and Nokkala 2003
			16+XY	*Nabis ericetorum* Scholtz, 1947 (Mikolajski 1965, 1967) *Nabis rugosus* (Linnaeus, 1758) (Schachow 1932, Leston 1957, Mikolajski 1965, 1967, Kuznetsova and Maryańska-Nadachowska 2000)
			18+XY	*Nabis ericetorum* (Leston 1957) *Nabis rugosus* (Leston 1957)
		*Nabis* (*Aspilaspis*) Stål, 1873 (3)	32+XY	Montgomery 1901a, Schachow 1932, Leston 1957, Mikolajski 1964, 1965, 1967, Takenouchi and Muramoto 1967, 1968, 1969, Muramoto 1979, Nokkala and Nokkala 1984b, Kuznetsova and Maryańska-Nadachowska 2000, Kuznetsova et al. 2004
		*Nabis* (*Dolichonabis*) Reuter, 1908 (2)	16+XY	
		*Nabis* (*Halonabis*) Reuter, 1890 (1)	32+XY	
		*Nabis* (*Limnonabis*) Kerzhner, 1968 (1)	16+XY	
		*Nabis* (*Milu*) Kirkaldy, 1907 (1)	16+XY	
		*Nabis* (*Nabicula*) Kirby, 1837 (2)	16+XY	
		*Nabis* (*Reduviolus*) Kirby, 1837 (1)	16+XY	
		*Nabis* (*Tropiconabis*) Kerzhner, 1968 (1)	16+XY	
	Prostemmatinae Reuter, 1890 (2/2)	*Pagasa* Stål, 1862 (1)	26+XY	Kuznetsova and Maryańska-Nadachowska 2000
		*Prostemma* Laporte, 1832 (1)	26+XY	Kuznetsova et al. 2004
**Superfamily Cimicoidea**				
**Anthocoridae Fieber, 1836** (3/5)	Anthocorinae s.str. Fieber, 1836 (2/4)	*Anthocoris* Fallén, 1814 (3)	28+XY	Southwood and Leston 1959*, Nokkala and Nokkala 1984b
		*Orius* Wolff, 1811 (1)	22+XY	Takenouchi and Muramoto 1971
	Xylocorinae Reuter, 1884 (1/1)	*Amphiareus* Distant, 1904 (1)	30+XY	Takenouchi and Muramoto 1971
**Cimicidae Latreille, 1802** (20/53)	Afrocimicinae Usinger, 1966 (1/1)	*Afrocimex* Schoutedon, 1951 (1)	22+X_1_X_2_Y	Ueshima 1966b
	Cacodminae Kirkaldy, 1899 (6/9)	*Aphrania* Ferris and Usinger, 1957 (1)	8+XY	Ueshima 1966b
		*Cacodmus* Stål, 1873 (2)	8+XY 10+XY	Ueshima 1966b Ueshima 1979
		*Crassicimex* Ferris and Usinger, 1957 (1)	36+X_1_X_2_Y	Ueshima 1966b
		*Leptocimex* Roubaud, 1913 (2)	22+XY	Ueshima 1966b, 1979
		*Loxaspis* Rothschild, 1912 (1)	8+XY	Ueshima 1966b
		*Stricticimex* Ferris and Usinger, 1957 (2)	22+XY (1) 36+X_1_X_2_Y (1)	Ueshima 1966b Ueshima 1979
	Cimicinae Latreille, 1802 (3/29)	*Cimex* Linnaeus, 1758 (19)	22+XY (1) 24+XY (1) 28+XY (1) 26+X_1_X_2_Y (3) 28+X_1_X_2_Y (6) 28+X_1_X_2_X_3_Y (6) 28+X_1_X_2_X_3_X_4_Y (1)	Slack 1938a, 1939a, b, Darlington 1939*, Ueshima 1963a, 1966b, 1968a, Grozeva and Nokkala 2002, Grozeva et al. 2010
		*Oeciacus* Stål, 1873 (2)	28+X_1_X_2_Y	Ueshima 1966b
		*Paracimex* Kiritschenko, 1914 (8)	36+X_1_X_2_Y (4) 36+X_1_X_2_ X_3_Y (2) 36+4-9XY (2)	Ueshima 1966b, 1968b
	Haematosiphoninae Jordan and Rothschild, 1912 (6/9)	*Acanthocrios* Del Ponte and Riesel, 1945 (1)	32+XY	*Acanthocrios furnarii* (Cordero and Vogelsang, 1928) (Ueshima 1966b: as *Caminicimex* Usinger, 1966)
			10+XY	*Acanthocrios furnarii* (Poggio et al. 2009)
		*Haematosiphon* Champion, 1900 (1)	28+X_1_X_2_Y	Ueshima 1966b
		*Hesperocimex* List, 1925 (3)	38+X_1_X_2_X_3_Y (1) 38+XY (1) 40+XY (1)	Ryckman and Ueshima 1964
		*Ornithocoris* Pinto, 1927 (2)	8+XY	Ueshima 1966b
		*Psitticimex* Usinger, 1966 (1)	28+X_1_X_2_Y	Ueshima 1966b, Poggio et al. 2009
		*Synxenoderus* List, 1925 (1)	28+X_1_X_2_Y	Ueshima 1966b
	Latrocimicinae Usinger, 1966 (2/3)	*Latrocimex* Lent, 1941(1)	22+XY	Ueshima 1966b
		*Leptocimex* Roubaud, 1913 (2)	22+XY	Ueshima 1966b, 1979
	Primicimicinae Ferris and Usinger, 1955 (2/2)	*Bucimex* Usinger, 1963 (1)	26+XY	Ueshima 1966b
		*Primicimex* Barber, 1941 (1)	28+XY	Ueshima 1968a
**Polyctenidae Westwood, 1874** (2/3)	Hesperocteninae Maa, 1964 (1/2)	*Hesperoctenes* Kirkaldi, 1906 (2)	4+XY (1) 10+XY (1)	Ueshima 1979
	Polycteninae Westwood, 1874 (1/1)	*Eoctenes* Kirkaldy, 1906 (1)	6+XY	Ueshima 1979

* In the paper, only the number of chromosomes (2n/n) is provided, then, the karyotype formula for the species is deduced here from 2n/n, ** But see the text

## References

[B1] Akingbohungbe AE (1974) Chromosome numbers of some numbers of some North American mirids (Heteroptera: Miridae). Canadian Journal of Genetics and Cytology 16: 251-256.

[B2] Angus RB Kemeny CK Wood EL (2004) The C-banded karyotypes of the four British species of *Notonecta* L. (Heteroptera: Notonectidae). Hereditas 140: 134-138. 10.1111/j.1601-5223.2004.01815.x 15061791

[B3] Ayala FJ Coluzzi M (2005) Chromosome speciation: humans, *Drosophila*, and mosquitoes. The Proceedings of the National Academy of Sciences of the United States of America (PNAS) 102: 6535-6542. doi:10.1073/pnas.0501847102 15851677 10.1073/pnas.0501847102 PMC1131864

[B4] Banerjee MK (1958) A study of the chromosomes during meiosis in twenty-eight species of Hemiptera (Heteroptera, Homoptera). Proceedings of Zoological Society (Calcutta) 11: 9-37.

[B5] Bardella VB Gaeta ML Vanzela ALL Azeredo-Oliveira MTV (2010) Chromosomal location of heterochromatin and 45S rDNA sites in four South American triatomines (Heteroptera: Reduviidae). Comparative Cytogenetics 4: 141-149. 10.3897/compcytogen.v4i2.50

[B6] Bargues MD Mera y Sierra RL Gomez HG Artigas P Mas-Coma S (2006) Ribosomal DNA ITS-1 sequencing of *Gabra truncatula* (Gastropoda, Lymnaeidae) and its potential impact on fascioliasis transmission in Mendoza, Argentina. Animal Biodiversity and Conservation 29: 191-194.

[B7] Barth R (1956) Estudos anatomicos e histologicos sobre a subfamilia Triatominae (Hemiptera: Reduviidae). VI. Estudo comparative sobre a espermiocitogenese das especies mais importantes. Memórias do Instituto Oswaldo Cruz. 54: 599-624. 10.1590/S0074-02761956000300009 13451161

[B8] Beukeboom LW (1994) Bewildering Bs: an impression of the 1st B-chromosome conference. Heredity 73: 328-336. 10.1038/hdy.1994.140

[B9] Blackman RL (1995) Sex determination in insects. In Leather SR, Hardie J (Eds) Insect Reproduction, CRC Press, Boca Raton, 57–94.

[B10] Bressa MJ Papeschi AG Mola LM Larramendy ML (1998) Meiotic studies in *Largus* *rufipennis* (Castelnau) (Largidae, Heteroptera) II Reciprocal translocation heterozygosity. Heredity 51: 253-264.

[B11] Camacho JPM (Ed) (2004) B Chromosomes in the Eukaryote Genome. Karger, Basel. 269 pp.

[B12] Camacho JPM Sharbel TF Beukeboon LW (2000) B-chromosome evolution. Philosophical Transactions of the Royal Society B: Biological Sciences 355: 163-178. 10.1098/rstb.2000.0556 PMC1692730 10724453

[B13] Carvalho AB Leonardo B Koerich LB Clark AG (2009) Origin and evolution of Y chromosomes: *Drosophila* tales. Trends in Genetics 25: 270-277. doi:10.1016/j.tig.2009.04.002 19443075 10.1016/j.tig.2009.04.002 PMC2921885

[B14] Cassis G Schuh RT (2010) Systematic methods, fossils, and relationships within Heteroptera (Insecta). Cladistics 26: 262-280. doi:10.1111/j.1096-0031.2009.00283.x 10.1111/j.1096-0031.2009.00283.x 34875785

[B15] Cobben RH (1986) A Most strikingly myrmecomorphic Mirid from Africa, with some notes on ant-mimicry and chromosomes in Hallodapines (Miridae, Heteroptera). Journal of the New York Entomological Society 94: 194-204.

[B16] Cook LG (2000) Extraordinary and extensive karyotypic variation: A 48-fold range in chromosome number in the gall inducing scale insect *Apiomorpha* (Hemiptera: Coccoidea: Eriococcidae). Genome 43: 255-263. 10791813

[B17] Coyne JA Orr HA (2004) Speciation. Sinauer Associates, Sunderland, 545 pp.

[B18] Darlington CD (1939) The genetical and mechanical properties of the sex chromosomes. V. *Cimex* and Heteroptera. Journal of Genetics 39: 101-138. 10.1007/BF02982821

[B19] De Meijere JC (1930) Űber einige europäische Insecten, besonders günstig zum Studium Reifungsteilungen, nebst einigen Zussätzen zur azetocarminmethode. Zoologischer Anzeiger 88: 209-219.

[B20] Dey SK Wangdi T (1988) Chromosome number and sex chromosome system in forty-four species of Heteroptera. Chromosome Information Service 45: 5-8.

[B21] Ekblom T (1941) Chromosomenuntersuchungen bei *Salda littoralis* L., *Callocoris chenopodii* Fall., und *Mesovelia furcata* Muls. & Rey, sowie Studien über die Chromosomen bei verschiedenen Hemiptera-Heteroptera im Hinblick auf phylogenetische Betrachtungen. Chromosoma 2: 12-35. 10.1007/BF00325951

[B22] Frìas-Lasserre D (2010) A New Species and Karyotype variation in the Bordering Distribution of *Mepraia spinolai* (Porter) and *Mepraia gajardoi* Frìas et al. (Hemiptera: Reduviidae: Triatominae) in Chile and its Parapatric model of Speciation. Neotropical Entomology 39: 572-583. 10.1590/S1519-566X2010000400017 20877994

[B23] Frydrychová R Grossmann P Trubac P Vítková M Marec F (2004) Phylogenetic distribution of TTAGG telomeric repeats in insects. Genome 47: 163–78. http://dx.doi.org/10.1139/g03-100 10.1139/g03-100 15060613

[B24] Geitler L (1939) Das Heterochromatin der Geschlechtshchromosomes bei Heteropteren. Chromosoma 1: 197-229. 10.1007/BF01271631

[B25] Goldsmith WM (1916) Relation of the true nucleolus to the linen network in the growth period of *Pselliodes cinctus*. Biological Bulletin 31: 121-136. 10.2307/1536361

[B26] Grozeva SM (1991) Karyotypes and structure of the reproductive system in *Piesma* (Heteroptera, Piesmatidae). Entomological review 70: 157-166.

[B27] Grozeva S (2003) Karyotype of three endemic Mediterranean Miridae species (Heteroptera) from Bulgaria. Acta Zoologica Bulgarica 55: 53-59.

[B28] Grozeva S Nokkala S (1996) Chromosomes and their meiotic behavior in two families of the primitive infraorder Dipsocoromorpha (Heteroptera). Hereditas 125: 31-36. 10.1111/j.1601-5223.1996.t01-1-00031.x

[B29] Grozeva S Nokkala S (2001) Chromosome numbers, sex determining systems, and patterns of the C-heterochromatin distribution in 13 species of lace bugs (Heteroptera, Tingidae). Folia biologica (Kraków) 49: 29-41. 11732164

[B30] Grozeva S Nokkala S (2002) Achiasmatic male meiosis in *Cimex* sp. (Heteroptera, Cimicidae). Caryologia 55: 189-192.

[B31] Grozeva S Nokkala S (2003) C-heterochromatin and extra (B) chromosome distribution in six species of the *Nabis* (Heteroptera, Nabidae) with the modal male karyotype 2n = 16+XY. Folia biologica (Krakòw) 51: 13-21. 14686643

[B32] Grozeva S Simov N (2008a) Cytotaxonomy of two *Cremnocephalus* species (Heteroptera: Miridae). In: Grozeva S, Simov N (Eds) 2008 Advances in Heteroptera Research, Festschrift in Honour of 80th Anniversary of Michail Josifov*,* 171–179.

[B33] Grozeva S Simov N (2008b) Cytogenetic Studies of Bryocorinae Baerensprung, 1860 True Bugs (Heteroptera: Miridae). Acta Zoologica Bulgarica Suppl 2: 61-70.

[B34] Grozeva S Simov N (2009) Cytogenetic study of two species of *Dryophilocoris*: *Dryophilocoris* (*Camarocyphus*) *flavoquadrimaculatus* (De Geer, 1773) and *Dryophilocoris* (*Dryophilocoris*) *luteus* (Herrich-Schaeffer, 1836) (Insecta, Heteroptera, Miridae). Genetics and Breedings, 38 (1): 41-45.

[B35] Grozeva S Kuznetsova V Anokhin B (2010) Bed bug cytogenetics: karyotype, sex chromosome system, FISH mapping of 18S rDNA, and male meiosis in *Cimex lectularius* Linnaeus, 1758 (Heteroptera: Cimicidae). Comparative Cytogenetics 4: 151-160.

[B36] Grozeva S Kuznetsova V Anokhin B (2011) Karyotypes, male meiosis and comparative FISH mapping of 18S ribosomal DNA and telomeric (TTAGG)_n_ repeat in seven species of true bugs (Hemiptera: Heteroptera). Comparative Cytogenetics 5 (4): 355-374. 10.3897/compcytogen.v5i4.2307 24260641 PMC3833783

[B37] Grozeva S Kuznetsova V Nokkala S (2004) Patterns of chromosome banding in four nabid species (Heteroptera, Cimicomorpha, Nabidae) with high chromosome number karyotypes. Hereditas 140: 99-104. 10.1111/j.1601-5223.2004.01782.x 15061786

[B38] Grozeva S Nokkala S Simov N (2006) First evidence of sex chromosome pre-reduction in male meiosis in the Miridae bugs (Heteroptera). Folia biologica (Kraków) 54: 9-12. 10.3409/173491606777919166 17044253

[B39] Grozeva S Nokkala S Simov N (2009) Chiasmate male meiosis in six species of water bugs from infraorders Nepomorpha and Gerromorpha (Insecta: Heteroptera). Comparative Cytogenetics 3: 125-130. 10.3897/compcytogen.v3i2.19

[B40] Grozeva S Simov N Josifov M (2007) Karyotaxonomy of some European *Macrolophus* species (Heteroptera: Miridae). Mainzer Naturwissenschaftliche Archive, Beiheft 31: 81-87.

[B41] Grozeva S Simov N Nokkala S (2008) Achiasmatic male meiosis in three *Micronecta* species (Heteroptera: Nepomorpha: Micronectidae). Comparative Cytogenetics 2: 73-78.

[B42] Harley KLS Kassulke RS (1971) Tingidae for biological control of *Lantana camara* (Verbenaceae). Entomophaga 16: 384-410. 10.1007/BF02370921

[B43] Helenius O (1952) The mode of bivalent orientation in the Hemiptera. Hereditas 38: 420-424. 10.1111/j.1601-5223.1952.tb02935.x

[B44] Henking H (1891) Über Spermatogenese und deren Beziehung zur Eientwicklung bei *Pyrrhocoris apterus* L. Zeit schrift für wissenschaftliche Zoologie 51: 685-736.

[B45] Hipp AL (2007) Nonuniform processes of chromosome evolution in sedges (*Carex*: Cyperaceae). Evolution 61 (9): 2175-2194. 10.1111/j.1558-5646.2007.00183.x 17767589

[B46] Hipp AL Rothrock PE Whitkus R Weber JA (2010) Chromosomes tell half of the story: the correlation between karyotype rearrangements and genetic diversity in sedges, a group with holocentric chromosomes. Molecular Ecology 19: 3124-3138. 10.1111/j.1365-294X.2010.04741.x 20618902

[B47] Jande SS (1959a) Chromosome number and sex mechanism in twenty seven species of Indian Heteroptera. Research Bulletin (Natural Sciences) of the Panjab University 10: 215-217.

[B48] Jande SS (1959b) Chromosome number and sex mechanism in nineteen species of Indian Heteroptera. Research Bulletin (Natural Sciences) of the Panjab University 10: 415-417.

[B49] Jande SS (1960) Pre-reductional sex chromosomes in the family Tingidae (Gymnocerata-Heteroptera). Nucleus 3: 209-214.

[B50] Joachimiak A Kula A Śliwińska E Sobieszczańska A (2001) C-banding and nuclear DNA amount in six *Bromus* species. Acta Biolologica. Cracovensia. Series Botanica 43: 105-115.

[B51] Ituarte S Papeschi AG (2004) Achiasmatic male meiosis in *Tenagobia* (*Fuscagobia*) *fuscata* (Stål) (Heteroptera, Corixoidea, Micronectidae). Genetica 122: 199-206. 10.1023/B:GENE.0000041048.75715.68 15609577

[B52] Jones RN Rees H (1982) B Chromosomes. Academic Press, 266 pp.

[B53] Jones N Houben A (2003) B chromosomes in plants: escapees from the A chromosome genome? Trends in Plant Science 8: 417–423. 10.1016/S1360-1385(03)00187-0 13678908

[B54] Kerzhner IM (1996) Family Nabidae A. Costa 1983 – damsel bugs. In: Aukema B Rieger C (Eds). 2*.* Amsterdam: 84-107.

[B55] King M (1993) Species Evolution. The Role of Chromosome Change. Cambridge University Press, 336 pp.

[B56] Kumar R (1971) Chromosomes of cocoa capsids (Heteroptera: Miridae). Caryologia 24: 229-237.

[B57] Kuznetsova VG Grozeva S (2008) Cytogenetic characters of *Arachnocoris trinitatus* Bergroth, 1916 (Insecta: Heteroptera: Nabidae) from nests of the spider *Coryssocnemis simla* Huber, 2000 (Aranea: Pholcidae). Comparative Cytogenetics 2: 139-142.

[B58] Kuznetsova VG Grozeva S (2010) Achiasmatic meiosis: a review. The Herald of Vavilov Society for Geneticists and Breeding Scientists 14: 79-88. [In Russian]

[B59] Kuznetsova V Grozeva S Nokkala S (2004) New cytogenetic data on Nabidae (Heteroptera, Cimicomorpha), with discussion on karyotype variation and meiotic patterns and their taxonomic significance. European Journal of Entomology 101: 205-210.

[B60] Kuznetsova VG Maryańska-Nadachowska A (2000) Autosomal polyploidy and male meiotic pattern in the bug family Nabidae. Journal of Zoological Systematics and Evolutionary Research 38: 87-94. 10.1046/j.1439-0469.2000.382131.x

[B61] Kuznetsova VG Grozeva S Sewlal J-N Nokkala S (2007) Cytogenetic characterization of the endemic of Trinidad, *Arachnocoris trinitatus* Bergroth: the first data for the tribe Arachnocorini (Heteroptera: Cimicomorpha: Nabidae). Folia biologica (Kraków) 55: 17-26. 10.3409/173491607780006344 17687930

[B62] Kuznetsova VG Nokkala S Shcherbakov DE (2002) Karyotype, reproductive organs, and pattern of gametogenesis in *Zorotypus hubbardi* Caudell (Insecta: Zoraptera, Zorotypidae), with discussion on relationships of the order. Canadian Journal of Zoology 80: 1047-1054. 10.1139/z02-074

[B63] Leston D (1957) Cyto-taxonomy of Miridae and Nabidae (Hemiptera). Chromosoma 8: 609-616. 10.1007/BF01259522 13523741

[B64] Lukhtanov VA Kandul NP Plotkin JB Dantchenko AV Haig D Pierce NE (2005) Reinforcement of pre-zygotic isolation and karyotype evolution in *Agrodiaetus* butterflies. Nature 436 (issue 7049): 385–389. 10.1038/nature03704 16034417

[B65] Lukhtanov VA Kuznetsova VG (2009) Molecular and cytogenetic approaches to species diagnostics, systematics, and phylogenetics. Zhurnal obshei biologii 70: 415-437. [In Russian] 19891413

[B66] Lukhtanov VA Kuznetsova VG (2010) What genes and chromosomes say about the origin and evolution of insects and other arthropods. Russian Journal of Genetics 46: 1115-1121. 10.1134/S1022795410090279 21061630

[B67] Maldonado J (1990) Systematic catalogue of the Reduviidae of the world (Insecta: Heteroptera). Caribbean Journal of Science, Special Edition, University of Puerto Rico, Mayagüez, 1–694.

[B68] Manna GK (1950) Multiple sex-chromosome mechanism in a reduviid bug *Conorhinus rubrofasciatus* (de Geer). Proceedings of the Zoological Society of Bengal 3: 155-161.

[B69] Manna GK (1951) A study of the chromosomes during during meiosis meiosis in forty-three species of Indian Heteroptera. Proceedings of the Zoological Society (Bengal) 4: 1-116.

[B70] Manna GK (1984) Chromosomes in evolution in Heteroptera. In Sharma AK, Sharma A (Eds) Chromosomes in evolution of eukaryotic groups. CRC Press, Boca, 189–225.

[B71] Manna GK Deb-Mallick S (1981) Meiotic chromosome constitution in forty-one species of Heteroptera. Chromosome Information Service 31: 9-11.

[B72] McClung CE (1902) The accessory chromosome-sex determinant? Biological Bulletin 3: 43–84. 10.2307/1535527

[B73] Mikolajski M (1964) Multiple sex-chromosome mechanism in *Nabis* Lt. (Heteroptera, Nabidae). Zoologica Poloniae 14: 15-18.

[B74] Mikolajski M (1965) Chromosome numbers in *Nabis* Lt. (Heteroptera, Nabidae). Experentia 21: 445. 10.1007/BF02150808 5870906

[B75] Mikolajski M (1967) Chromosome studies in the genus *Nabis* Lt. (Heteroptera, Nabidae). Zoologica Poloniae 17: 323-343

[B76] Montgomery TH (1901a) A study of the chromosomes of the germ cells of Metazoa. Transactions of the American Philosophical Society 20: 154-236. 10.2307/1005428

[B77] Montgomery TH (1901b) Further studies on the chromosomes of the Hemiptera-Heteroptera. Proceedings of the Academy of Natural Sciences of Philadelphia 53: 261-271.

[B78] Montgomery TH (1906) Chromosomes and spermatogenesis of the Hemiptera-Heteroptera. Transactions of the American Philosophical Society 21: 97-173. 10.2307/1005443

[B79] Muramoto N (1973a) A chromosome study in eighteen Japanese Heteropterans. La Kromosomo 91: 2896-2905. [In Japanese]

[B80] Muramoto N (1973b) A study of the B-chromosome numbers and their occurrence rate in *Orthocephalus funestus* Jakovlev (Miridae; Heteroptera). La Kromosomo 91: 2906-2912.

[B81] Muramoto N (1973c) A list of the chromosome numbers of Heteropteran insects of Japan. CIS 14: 29-31.

[B82] Muramoto N (1974) Meiotic behaviour of chromosomes in *Orthocephalus funestus* Jakovlev (Miridae: Heteroptera). Chromosome Information Service 17: 23-24.

[B83] Muramoto N (1978) A chromosome study of thirty Japanese heteropterans (Heteroptera). Genetica 37–44.

[B84] Muramoto N (1979) A chromosome study of 15 species of Heteroptera from Australia and Japan. La Kromosomo 14: 390-397.

[B85] Nokkala S (1986a) The mechanisms behind the regular segregation of the m-chromosomes in *Coreus marginatus* L. (Coreidae, Hemiptera). Hereditas 105: 73-85. 10.1111/j.1601-5223.1986.tb00645.x

[B86] Nokkala S (1986b) The mechanisms behind the regular segregation of autosomal univalents in *Calocoris quadripunctatus* (Vil.) (Miridae, Hemiptera). Hereditas 105: 199-204. 10.1111/j.1601-5223.1986.tb00662.x

[B87] Nokkala S Grozeva S (2000) Male meiosis of achiasmatic type in *Myrmedobia coleoptrata* (Fn.) (Heteroptera, Microphysidae). Caryologia 53: 5-8.

[B88] Nokkala S Nokkala C (1983) Achiasmatic male meiosis in two species of *Saldula* (Saldidae, Hemiptera). Hereditas 99: 131-134. 10.1111/j.1601-5223.1983.tb00737.x 6643081

[B89] Nokkala S Nokkala C (1984a) The occurrence of the X0 sex chromosome system in *Dictyonota tricornis* (Schr.) (Tingidae, Hemiptera) and its significance for concepts of sex chromosome system evolution in Heteroptera. Hereditas 100: 299-301. 10.1111/j.1601-5223.1984.tb00130.x

[B90] Nokkala S Nokkala C (1984b) Achiasmatic male meiosis in the Heteropteran genus *Nabis* (Nabidae, Hemiptera). Hereditas 101: 31-35. 10.1111/j.1601-5223.1984.tb00445.x

[B91] Nokkala S Nokkala C (1986a) Achiasmatic male meiosis in *Anthocoris nemorum* (L.) (Anthocoridae, Hemiptera). Hereditas 105: 287-289. 10.1111/j.1601-5223.1986.tb00675.x

[B92] Nokkala S Nokkala C (1986b) Achiasmatic male meiosis of collochore type in the heteropteran family Miridae. Hereditas 105: 193-197. 10.1111/j.1601-5223.1986.tb00661.x

[B93] Nokkala S Nokkala C (1997) The absence of chiasma terminalization and inverted meiosis in males and females of *Myrmus miriformis* Fn. (Corizidae, Heteroptera). Heredity 78: 561-566. 10.1038/hdy.1997.87

[B94] Nokkala S Nokkala C (2004) Interaction of B chromosomes with A or B chromosomes in segregation in insects. Cytogenetic and Genome Research 106: 394-397. 10.1159/000079317 15292621

[B95] Nokkala S Grozeva S Kuznetsova V Maryańska-Nadachowska A (2003) The origin of the achiasmatic XY sex chromosome system in *Cacopsylla peregrina* (Frst.) (Psylloidea, Homoptera). Genetica 119: 327-332. 10.1023/B:GENE.0000003757.27521.4d 14686611

[B96] Nokkala C Kuznetsova V Grozeva S Nokkala S (2007) Direction of karyotype evolution in the bug family Nabidae (Heteroptera): New evidence from 18S rDNA analysis. European Journal of Entomology 104: 661-665.

[B97] Nokkala S Kuznetsova VG Maryanska-Nadachowska A Nokkala C (2006) Holocentric chromosomes in meiosis. II. The modes of orientation and segregation of a trivalent. Chromosome Research 14: 559-565. 10.1007/s10577-006-1053-6 16823618

[B98] Panzera F Dujardin JP Nicolini P Caraccio MN Rose V Tellez T Bermudez H Bargues MD Mas-Coma S O‘Connor JE Pérez R (2004) Genomic changes of Chagas disease vector, South America. Emerging Infectious Diseases 10: 438-446. 15109410 10.3201/eid1003.020812 PMC3322799

[B99] Panzera F Ferrandis I Ramsey J Ordocez R Salazar-Schettino PM Cabrera M Monroy MC Bargues MD Mas-Coma S O‘Connor JE Angulo VM Jaramillo N Cordón-Rosales C Gómez D Pérez R (2006) Chromosomal variation and genome size support existence of cryptic species of *Triatoma dimidiata* with different epidemological importance as Chagas disease vectors. Tropical Medicine and International Health 11: 1092-1103. 10.1111/j.1365-3156.2006.01656.x 16827710

[B100] Panzera F Perez R Panzera Y Alvarez F Scvortzoff E Salvatella R (1995) Karyotype evolution in holocentric chromosomes of three related species of triatomines (Hemiptera-Reduviidae). Chromosome Research 3: 143-150. 7780658 10.1007/BF00710707

[B101] Panzera F Pérez R Panzera Y Ferrandis I Ferreiro MJ Calleros L (2010) Cytogenetics and genome evolution in the subfamily Triatominae (Hemiptera, Reduviidae). Cytogenetic and Genome Research 128: 77-87. 10.1159/000298824 20407223

[B102] Papeschi AG Bressa MJ (2006a) Classical and molecular cytogenetics in Heteroptera. Research Advances in Entomology 1: 1-9.

[B103] Papeschi AG Bressa MJ (2006b) Evolutionary cytogenetics in Heteroptera. Journal of Biological Reaserch 5: 3-21.

[B104] Papeschi AG Mola LM (1990) Meiotic studies in *Acanonicus hahni* (Coreidae, Heteroptera). I. Behavior of univalents in desynaptic individuals. Genetica 80: 31-38. 10.1007/BF00120117

[B105] Paulmier FC (1899) The spermatogenesis of *Anasa tristis*. Journal of Morphology 15(Suppl): 224–272.

[B106] Payne F (1909) Some new types of chromosome distribution and their relation to sex. Biological Bulletin 16: 119-166. 10.2307/1536127

[B107] Payne F (1910) The chromosomes of *Acholla multispinosa*. Biological Bulletin 18: 174-179. 10.2307/1536011

[B108] Payne F (1912) I. A further study of the chromosomes of the Reduviidae. II. The nucleolus in the yang oocytes and the origin of the ova in *Gelastocoris*. Journal of Morphology 23: 331-347. 10.1002/jmor.1050230206

[B109] Pérez R Calleros L Rose V Lorca M Panzera F (2004) Cytogenetic studies on *Mepraia gajardoi* (Heteroptera: Reduviidae). Chromosome behaviour in a spontaneous translocation mutant. European Journal of Entomology 101: 211-218.

[B110] Pérez R Panzera F Page J Suja JA Rufas JS (1997) Meiotic behaviour of holocentric chromosomes: orientation and segregation of autosomes in *Triatoma infestans* (Heteroptera). Chromosome Research 5: 47-56. 10.1023/A:1018493419208 9088643

[B111] Petitpierre E (1996) Molecular cytogenetics and taxonomy of insects, with particular reference to the Coleoptera. Journal of Insect Morphology and Embryology 25: 115-133. 10.1016/0020-7322(95)00024-0

[B112] Piza SdT (1957) Comportamento dos cromossomios na espermatogenese de *Microtomus conspicillaris* (Drury). Revista de Agricultura 32: 53-64.

[B113] Poggio MG Bressa MJ and Papeschi AG (2007). Karyotype evolution in Reduviidae (Insecta: Heteroptera) with special reference to Stenopodinae and Harpactorinae. Comparative Cytogenetics 1: 159-168.

[B114] Poggio MG Bressa MJ Papeschi AG (2011) Male meiosis, heterochromatin characterization and chromosomal location of rDNA in *Microtomus lunifer* (Berg, 1900) (Hemiptera, Reduviidae, Hammacerinae). Comparative Cytogenetics 5: 1-22. 10.3897/compcytogen.v5i1.1143 24260616 PMC3833732

[B115] Poggio MG Bressa MJ Papeschi AG Di Iorio O (2009) Insects found in birds’ nests from Argentina: cytogenetic studies in Cimicidae (Hemiptera) and its taxonomical and phylogenetic implications. Zootaxa 2315: 39-46.

[B116] Putshkov VG Putshkov PV (1986–1989) A catalogue of the Reduviidae (Heteroptera) of the world, 6 volumes. Vinity, Lyubertsy.

[B117] Ryckman RE Ueshima N (1964) Biosystematics of the *Hesperocimex* complex (Hemiptera: Cimicidae) and avian hosts (Piciformes: Picidae: Passerformes: Hirundinidae). Annals of Entomological Society of America 57: 624-638.

[B118] Sahara K Marec F Traut W (1999) TTAGG telomeric repeats in chromosomes of some insects and other arthropods. Chromosome Research 7: 449-460. 10.1023/A:1009297729547 10560968

[B119] Satapathy SN Patnaik SC (1989) Chromosome numbers in forty-one species of Indian Heteroptera. Chromosome Information Service 47: 3-5.

[B120] Schachow F (1932) Abhandlungen über haploide Chromosomengarnituren in den Samendrüsen der Hemiptera. Anatomischer Anzeiger 75: 1-46.

[B121] Schreiber G Pellegrino J (1950) Eteropicnosi di autosomi come possible mecanismo di speciazione. Science Genetics 3: 215-226. 15431074

[B122] Schreiber G Bogliolo AN Coelho de Pinho A (1972) Cytogenetics of Triatominae: karyotype, DNA content, nuclear size and heteropycnosis of autosomes. Revista Brasileira de Biologia 32: 255-263.

[B123] Schuh RT (1995) Plant bugs of the world (Insecta: Heteroptera: Miridae). Systematic Catalog Distributions, Host list, and Bibliography. The New York Entomoogical Society, New York, 1329 pp.

[B124] Schuh RT (2011) On-line Systematic Catalog of Plant Bugs (Insecta: Heteroptera: Miridae) http://research.amnh.org/pbi/catalog/local/intro.php#about

[B125] Schuh RT Slater JA (1995) True Bugs of the World (Hemiptera: Heteroptera). Classification and Natural History. Cornell University Press, Ithaca, New York, xii + 336 pp.

[B126] Schuh RT Štys P (1991) Phylogenetic analysis of cimicomorphan family relationships (Heteroptera). Journal of the New York Entomological Society 99: 298-350.

[B127] Schuh RT Weirauch C Wheeler W (2009) Phylogenetic relationships within the Cimicomorpha. Systematic Entomology 34: 15-48. 10.1111/j.1365-3113.2008.00436.x

[B128] Schrader F (1940) The formation of tetrads and the meiotic mitoses in the male of *Rhytidolomia senilis* Say (Hemiptera, Heteroptera). Journal of Morphology 67: 123-141. 10.1002/jmor.1050670106

[B129] Schrader F (1947) The role of kinetochore in the chromosomal evolution of the Hemiptera and Homoptera. Evolution 1: 134-142. 10.2307/2405489

[B130] Severi-Aguiar GD Lourenco LB Bicudo HE Azeredo-Oliveira MT (2006). Meiosis aspects and nucleolar activity in *Triatoma vitticeps* (Triatominae, Heteroptera). Genetica 126: 141-151. 10.1007/s10709-005-1443-2 16502091

[B131] Slack HD (1938a) Chromosome numbers in *Cimex*. Nature 142: 358. 10.1038/142358a0

[B132] Slack HD (1938b) Cytogenetic studies on five families of Hemiptera-Heteroptera. Thesis, University of Edinbugh.

[B133] Slack HD (1939a) The chromosomes of *Cimex*. Nature 143: 78. 10.1038/143078a0

[B134] Slack HD (1939b) Structural hybridity in *Cimex* L. Chromosoma 1: 104-118. 10.1007/BF01271624

[B135] Southwood TRE Leston D (1959) Land and water bugs of the British Isles. Frederick Warne and Co Ltd, London, 436 pp.

[B136] Takenouchi Y Muramoto N (1967) A survey of the chromosomes in twenty species of Heteroptera insects. Journal of Hokaido University of Education II B 18: 1-15. [In Japanese]

[B137] Takenouchi Y Muramoto N (1968) A survey of the chromosomes in twenty three species of Heteroptera insects. Journal of Hokkaido University of Education II B 19: 1-19. [in Japanese]

[B138] Takenouchi Y Muramoto N (1969) Chromosomes numbers of Heteroptera. Journal of Hokkaido University of Education II B 20: 1-15.

[B139] Takenouchi Y Muramoto N (1970) A study of chromosomes in five species of Heterotperan Insects. Journal of Hokkaido University of Education II B 21: 9-13. [In Japanese]

[B140] Takenouchi Y Muramoto N (1971) A study of the chromosomes in three species of heteropteran insects (Anthocoridae and Veliidae: Heteroptera). Journal of Hokkaido University of Education II B 22: 23-25. [In Japanese]

[B141] Takenouchi Y Muramoto N (1972a) A survey of the chromosomes of three species of the genus *Adelphocoris* (Miridae, Heteroptera). Kontyu 40: 132-136. [In Japanese]

[B142] Takenouchi Y Muramoto N (1972b) A survey of the fragment chromosome in *Orthocephalus funestus* Jakovlev (Miridae: Heteroptera). Oyo-dobutsugaku-zasshi 5: 218-224. [In Japanese]

[B143] Thomas DB (1996) The role of polyploidy in the evolution of the Heteroptera. In: Schaefer CW (Ed). Studies of Hemipteran Phylogeny. Entomological Society of America, Lanham: 159-178.

[B144] Toshioka S (1933) On the chromosomes in Hemiptera-Heteroptera. I. Chromosomes in *Velinas nodipes* Uhler. Oyo-dobutsugaku-zasshi 6: 109-115. [In Japanese]

[B145] Toshioka S (1934) On the chromosomes in Hemiptera-Heteroptera. II. Oyo-dobutsugaku-zasshi 6: 34-42. [In Japanese]

[B146] Toshioka S (1936) On the chromosomes types in several families of Heteroptera. (in Japanese). Oyo-dobutsugaku-zasshi 8: 167-168. [In Japanese]

[B147] Troedsson PH (1944) The behaviour of the compound sex chromosomes in the females of certain Hemiptera-Heteroptera. Journal of Morphology 75: 103-147. 10.1002/jmor.1050750106

[B148] Ueshima N (1963a) Chromosome behaviour of the *Cimex pilosellus* complex. (Cimicidae-Hemiptera). Chromosoma 14: 511-521. 10.1007/BF00321470 14096526

[B149] Ueshima N (1963b) Chromosome cytology of two species in Emesinae (Reduviidae:Hemiptera). CIS 4: 12-14.

[B150] Ueshima N (1966a) Cytotaxonomy of the Triatominae (Reduviidae, Hemiptera). Chromosoma 18: 97-122. 10.1007/BF00326447

[B151] Ueshima N (1966b) Cytology and cytogenetics (pp. 183–237). In: Usinger R.L. (Ed.), Monograph of Cimicidae, New York, 585 pp.

[B152] Ueshima N (1968a) Cytolology and bionomics of *Primicimex* *cavernis* Barber (Cimicidae; Hemiptera). The Pan Pacific Entomologist 44: 145-152.

[B153] Ueshima N (1968b) Distribution, host relationships and speciation of the genus *Paracimex* (Cimicidae: Hemiptera). Mushi 42: 15-27.

[B154] Ueshima N (1979) Hemiptera II: Heteroptera. In: John B (Ed) Animal Cytogenetics. 3. Insecta 6, Gebrűder Borntraeger, Berlin, Stuttgart, 113 pp.

[B155] Viera A Page J Rufas JS (2009) Inverted meiosis: The true bugs as a model to study. In: R Benavente Volff J-N (Eds). Meiosis. Genome Dynamics. Basel, Karger 5: 137–156. 10.1159/000166639 18948713

[B156] Wang Y-P Bu W-J Zhang H-F (2003) On the karyotype of *Anthocoris montanus* Zheng (Heteroptera, Anthocoridae). Acta Zootaxonomica Sinica 28: 126-129.

[B157] Weirauch C (2008) Cladistic analysis of Reduviidae (Hetroptera, Cimicomorpha) based on morphological characters. Systematic Enthomology 33: 229-274. 10.1111/j.1365-3113.2007.00417.x

[B158] Wheeler AG (2001) Biology of the plant bugs (Hemiptera: Miridae): pests, predators, opportunists. Cornell University Press, 507 pp.

[B159] Wheeler WC Schuh RT Bang R (1993) Cladistic relationships among higher groups of Heteroptera: congruence between morphological and molecular data sets. Entomologica Scandinavica 24: 121-37. 10.1163/187631293X00235

[B160] White MJD (1973) Animal cytogenetics and evolution. Cambridge University Press, Cambridge, 961 pp.

[B161] White MJD (1978) Modes of speciation. San Francisco: W.H. Freeman and Company, 455 pp.

[B162] Wilson EB (1905) The chromosomes in relation to the determination of sex in insects. Science 22: 500-502. 17748139 10.1126/science.22.564.500

[B163] Wilson EB (1909) Studies on chromosomes. V. The chromosomes of *Metapodius*. A contribution to the hypothesis of the genetic continuity of chromosomes. Journal of Experimental Zoology 6: 147-205. 10.1002/jez.1400060202

[B164] Wilson EB (1925) The Cell in Development and Heredity, 3rd edition. Macmillan, New York, 923 pp.

[B165] Wolf KW (1996) The structure of condensed chromosomes in mitosis and meiosis of insects. International Journal of Insect Morphology and Embryology 25: 37-62. 10.1016/0020-7322(95)00021-6

[B166] Yoshida TH (1947) A chromosome survey in 20 species of Heteropteran insects, with special reference to the morphology of sex chromosomes. II La Kromosomo 3–4: 139–147. [In Japanese]

[B167] Yoshida TH (1950) A chromosome survey in 12 species of Hemiptera. Iden-no-sogokenkyu 1: 85-91. [In Japanese]

[B168] Zakian VA (1995) Telomeres: Beginning to Understand the End. Science 270: 1601-1607. 10.1126/science.270.5242.1601 7502069

